# A Potential Peptide Therapeutic Derived from the Juxtamembrane Domain of the Epidermal Growth Factor Receptor

**DOI:** 10.1371/journal.pone.0049702

**Published:** 2012-11-15

**Authors:** Aislyn D. W. Boran, Joseph Seco, Vinodh Jayaraman, Gomathi Jayaraman, Shan Zhao, Sushmitha Reddy, Yibang Chen, Ravi Iyengar

**Affiliations:** Department of Pharmacology and Systems Therapeutics, Mount Sinai School of Medicine, New York, New York, United States of America; University of Illinois at Chicago, United States of America

## Abstract

The epidermal growth factor receptor (EGFR) is involved in many cancers and EGFR has been heavily pursued as a drug target. Drugs targeting EGFR have shown promising clinical results for several cancer types. However, resistance to EGFR inhibitors often occurs, such as with KRAS mutant cancers, therefore new methods of targeting EGFR are needed. The juxtamembrane (JXM) domain of EGFR is critical for receptor activation and targeting this region could potentially be a new method of inhibiting EGFR. We hypothesized that the structural role of the JXM region could be mimicked by peptides encoding a JXM amino acid sequence, which could interfere with EGFR signaling and consequently could have anti-cancer activity. A peptide encoding EGFR 645–662 conjugated to the Tat sequence (TE-64562) displayed anti-cancer activity in multiple human cancer cell types with diminished activity in non-EGFR expressing cells and non-cancerous cells. In nude mice, TE-64562 delayed MDA-MB-231 tumor growth and prolonged survival, without inducing toxicity. TE-64562 induced non-apoptotic cell death after several hours and caspase-3-mediated apoptotic cell death with longer treatment. Mechanistically, TE-64562 bound to EGFR, inhibited its dimerization and caused its down-regulation. TE-64562 reduced phosphorylated and total EGFR levels but did not inhibit kinase activity and instead prolonged it. Our analysis of patient data from The Cancer Genome Atlas supported the hypothesis that down-regulation of EGFR is a potential therapeutic strategy, since phospho- and total-EGFR levels were strongly correlated in a large majority of patient tumor samples, indicating that lower EGFR levels are associated with lower phospho-EGFR levels and presumably less proliferative signals in breast cancer. Akt and Erk were inhibited by TE-64562 and this inhibition was observed *in vivo* in tumor tissue upon treatment with TE-64562. These results are the first to indicate that the JXM domain of EGFR is a viable drug target for several cancer types.

## Introduction

The epidermal growth factor receptor (EGFR/ErbB1), a member of the ErbB family of receptor tyrosine kinases, is amplified or over-active in many types of epithelial cancers, including pancreatic cancer, breast cancer, brain cancer, non-small cell lung cancer, colorectal cancer, breast and head and neck squamous-cell carcinoma [1], [2], [3]. Aberrant EGFR signaling in cancer is involved in increased tumor cell proliferation and growth rates, anchorage independent growth and metastasis formation [3]. Due to its role in cancer cell progression and survival, several anti-cancer therapies target EGFR [1], [4] have been approved by the FDA. Anti-EGFR therapies can be classified into two general types: tyrosine kinase inhibitors (TKIs), such as gefitinib and erlotinib, which inhibit the kinase domain and monoclonal antibodies which inhibit the extracellular ligand-binding domain, such as cetuximab. The anti-EGFR therapies have displayed promising activity in the clinic in certain cancer types; however, there are issues with intrinsic and acquired resistance [1], [2]. For instance, colorectal tumors and lung tumors, which exhibit mutations in KRAS, are much more likely to be resistant to cetuximab [3], [5] and to gefitinib and erlotinib [6], [7], respectively. In an example of acquired drug resistance in lung cancer, chronic gefitinib treatment leads to tumors which express a mutant form of EGFR, which has reduced affinity for the drug [3], [8], [9]. Given the multiplicity of the resistance mechanisms to EGFR therapies, new approaches to targeting EGFR are important to cancer drug discovery.

We propose that the juxtamembrane (JXM) domain of EGFR is a new region that could serve as a drug target. Recent studies have shown that the JXM domain of EGFR is critical for intrinsic tyrosine kinase activity [10], [11], [12]. In the presence of the JXM domain, EGFR kinase activity is 70-fold higher compared to the intracellular domain alone [13]. Also, the JXM domain mediates the allosteric regulation of EGF binding EGFR [11] and the interaction of EGFR with phosphatidylinositol 4,5-biphosphate and Ca^++^/calmodulin at the membrane [14], [15], [16], [17]. The recently reported structure of the full intracellular domain of EGFR showed [18] that the JXM region makes two major areas of contact in the active, asymmetrical dimer [19]. The structurally distinct EGFR JXM regions are called the JMA and JMB regions. The JMB region creates a “latch” by hooking over onto the kinase domain of the opposite monomer. Two helical JMA segments, one from each monomer, interact with one another in an anti-parallel manner, forming a helical dimer [13].

If the interactions of the JXM region of EGFR could be mimicked by peptides encoding the JXM amino acid sequence, then these peptides could potentially interfere with EGFR signaling which is often related to cell survival and proliferation. In support of this hypothesis, two previous studies have shown that ErbB signaling was inhibited with peptides derived from the transmembrane domain [20], [21]. One study showed that ErbB transmembrane receptor fragments could mitigate receptor signaling through dimerization inhibition [20]. Another study showed that ErbB2 transmembrane peptides or short proteins prevented receptor dimerization and inhibited function and slowed growth of transformed cells, colonies and tumors [21]. These studies suggest that novel methods of inhibiting ErbB receptors may exist and should be exploited as cancer therapies. Hence, we hypothesized that peptides encoding the EGFR JXM region could have anti-cancer activity.

We assayed peptides from the JXM region for anti-cancer properties and for their ability to modulate EGFR signaling. One peptide from the JMA region, which we designated as TE-64562, displayed anti-cancer activity in human cancer cells from different tissues and in a MDA-MB-231 breast cancer xenograft model. TE-64562 induced activation of stress signaling (JNK and p38) which resulted in multiple modes of cell death. EGFR plays a role in cellular stress signaling which has been associated with its down-regulation [22], [23] and has been shown to induce both non-apoptotic and apoptotic cell death in cardiomyocytes [24]. TE-64562 bound to EGFR at the JXM region, inhibited its dimerization, caused its down-regulation and prolonged its phosphorylation. TE-64562 inhibited downstream EGFR signaling at Erk and Akt in MDA-MB-231 cells and *in vivo*, in tumors upon intraperitoneal administration. Taken together, these results indicate that the juxtamembrane domain of EGFR is a viable drug target for several cancers.

## Results

### Design of EGFR JXM Region Peptides and Assessment of Activity in Cell Viability Assay

**Figure 1:**
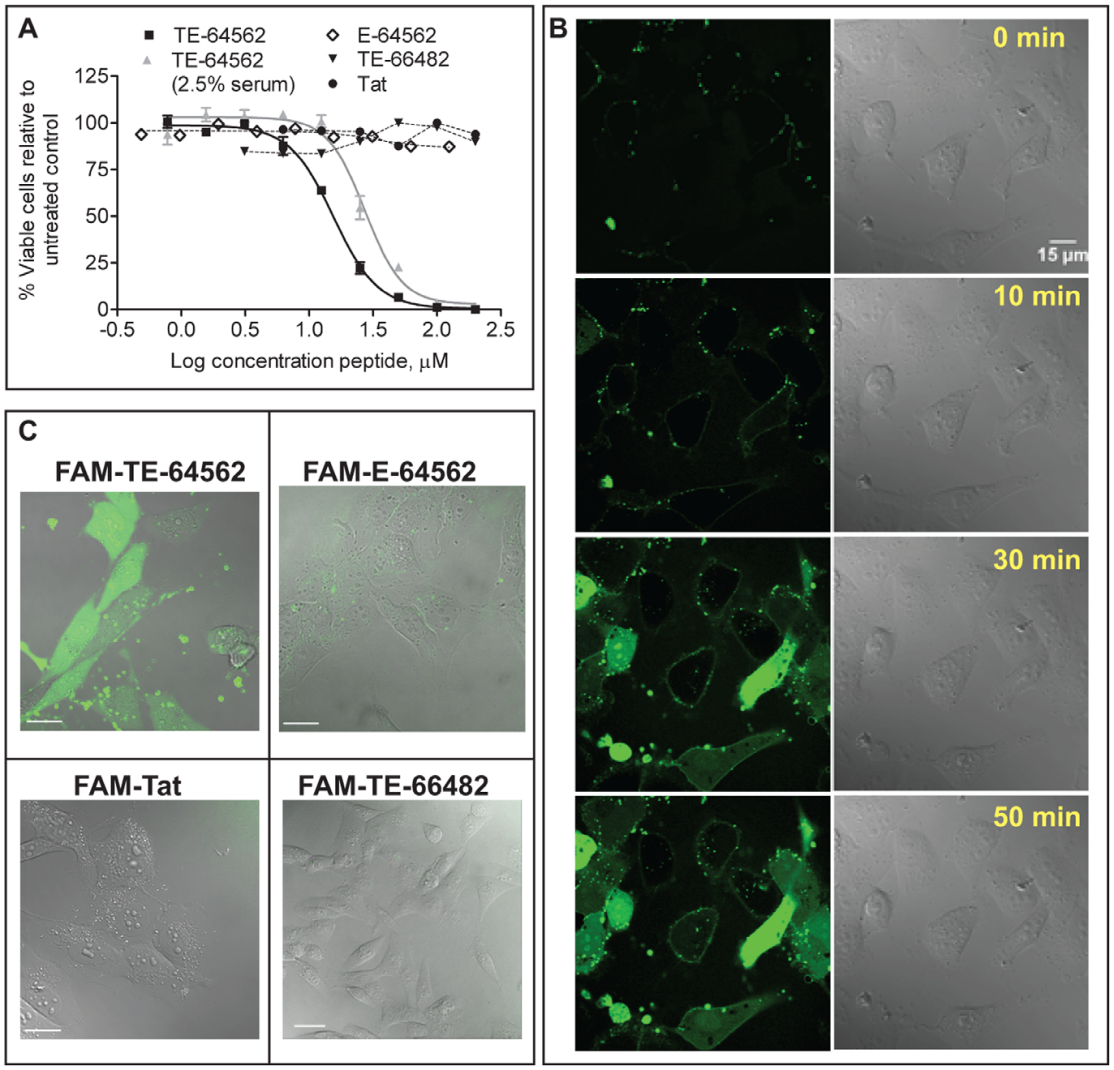
Fluorescent confocal microscopy and activity of EGFR juxtamembrane peptides and control peptides in MDA-MB-231 cells. (**A**) The Tat-conjugated EGFR JMA peptide (TE-64562) was added to the MDA-MB-231 cells, in 2.5% serum (gray triangles) or serum free media (black squares). The non-Tat-conjugated EGFR JMA peptide (E-645-662, diamonds), the Tat peptide (Tat, black circles) or Tat-conjugated EGFR JMB peptide (TE-66482, black inverted triangles) were added to the MDA-MB-231 cells in serum free media. The cell viability, measured after overnight treatment, is plotted. The plot represents one of three independent experiments, run in triplicate. Error bars represent standard error of the mean of triplicate values from one experiment. (**B–D**) Peptides were N-terminally labeled with 5-carboxyfluorescein (FAM). (**B**) MDA-MB-231 cells were treated with FAM-TE-64562 (1.25 µM) for the indicated amounts of time. Scale bar = 15 µm. (**C**) Cells were treated overnight with FAM-TE-64562 (5.0 µM), FAM-645-662 (5.0 µM), FAM-Tat (2.5 µM) or FAM-664-682 (5.0 µM) and imaged the following day. Scale bars are 40 µm. Also see Figure S1.

In order to test both regions [13], [18] of the EGFR JXM domain (JMA and JMB), we designed peptides encoding the EGFR JMA region (645–662) and the JMB region (663–682). We tested the activity in a cell viability assay in MDA-MB-231 cells, which express a high level of EGFR [25]. Since peptides often require a carrier for cellular entry, we conjugated the JMA and JMB sequences to the human immunodeficiency virus (HIV) transactivator of transcription (Tat) sequence (HIV Tat 49–58, Table 1), a known cargo carrier of proteins/peptides across the cellular membrane [26]. The Tat-conjugated 645–662 peptide (TE-64562) displayed an EC_50_ of 12.6±2.3 µM in a cell viability assay of serum starved MDA-MB-231 cells (Table 1 and Fig. 1A), which was reduced in the presence of serum (Fig. 1A and S2). The 645–662 peptide (without Tat; E-64562) and the Tat-conjugated JMB peptide (TE-64582) did not display any activity up to 200 µM (Table 1 and Fig. 1A). Control peptides were generated with the Tat sequence alone (Tat), the EGFR JMA sequence with the positive charged amino acids maintained and alanines inserted at all other positions (T-poly-Ala), and the EGFR JMA sequence with charged amino acids switched to amino acids with opposite charge (T-control) (Table 1). These control peptides did not have any effect on the viability of MDA-MB-231 cells (Table 1). Of the peptides tested, the TE-64562 peptide displayed the most robust activity at reducing cell viability of MDA-MB-231 breast cancer cells and was therefore further characterized.

**Table 1:** Activity of EGFR juxtamembrane peptides in viability assay of MDA-MB-231 cells.

Name	Sequence	EC_50_± S.D.^a^(µM)
Tat	RKKRRQRRRG	>100
E-64562	RRRHIVRKRTLRRLLQER	>100
**TE-64562**	**Tat-RRRHIVRKRTLRRLLQER**	**12.6±2.3**
TE-66482	Tat-VEPLTPSGEAPNQALLRI	>100
T-poly-Ala	Tat-RRRHAARKRAARRLLAAR	>100
T-control	Tat-EERARVEERALEELRIEE	>100

a Values are shown with standard deviation values (S.D.) from at least three independent experiments.

### Cellular Entry Kinetics of EGFR JXM Peptides in MDA-MB-231 Cells

To establish whether Tat-conjugation was necessary for cellular entry, the Tat, TE-64562, E-64562 and TE-66482 peptides were N-terminally labeled with 5-carboxyfluorescein (FAM) and monitored using live-cell fluorescent confocal microscopy in MDA-MB-231 cells (Fig. 1B, 1C and Fig. S1). The TE-64562 peptide (2.5 µM) entered cells after approximately 10 minutes, initially accumulated at the membrane and then became distributed throughout the cell while maintaining some membrane localization (Fig. 1B). Cells treated with the FAM-conjugated E-64562 peptide (without Tat) did not display any fluorescence within the interior of the cell when monitored up to overnight incubation (Fig. 1C). Therefore, Tat-conjugation is necessary to facilitate cellular entry of the 645–662 JMA sequence. MDA-MB-231 cells treated with the FAM-conjugated Tat peptide or the FAM-labeled TE-66482 peptide did not display any fluorescence within the interior of the cell when monitored up to 90 minutes (Fig. S1) or after overnight incubation (Fig. 1C).

### The TE-64562 Peptide Inhibits Viability of Human Cancer Cell Lines from Different Tissues

**Figure 2:**
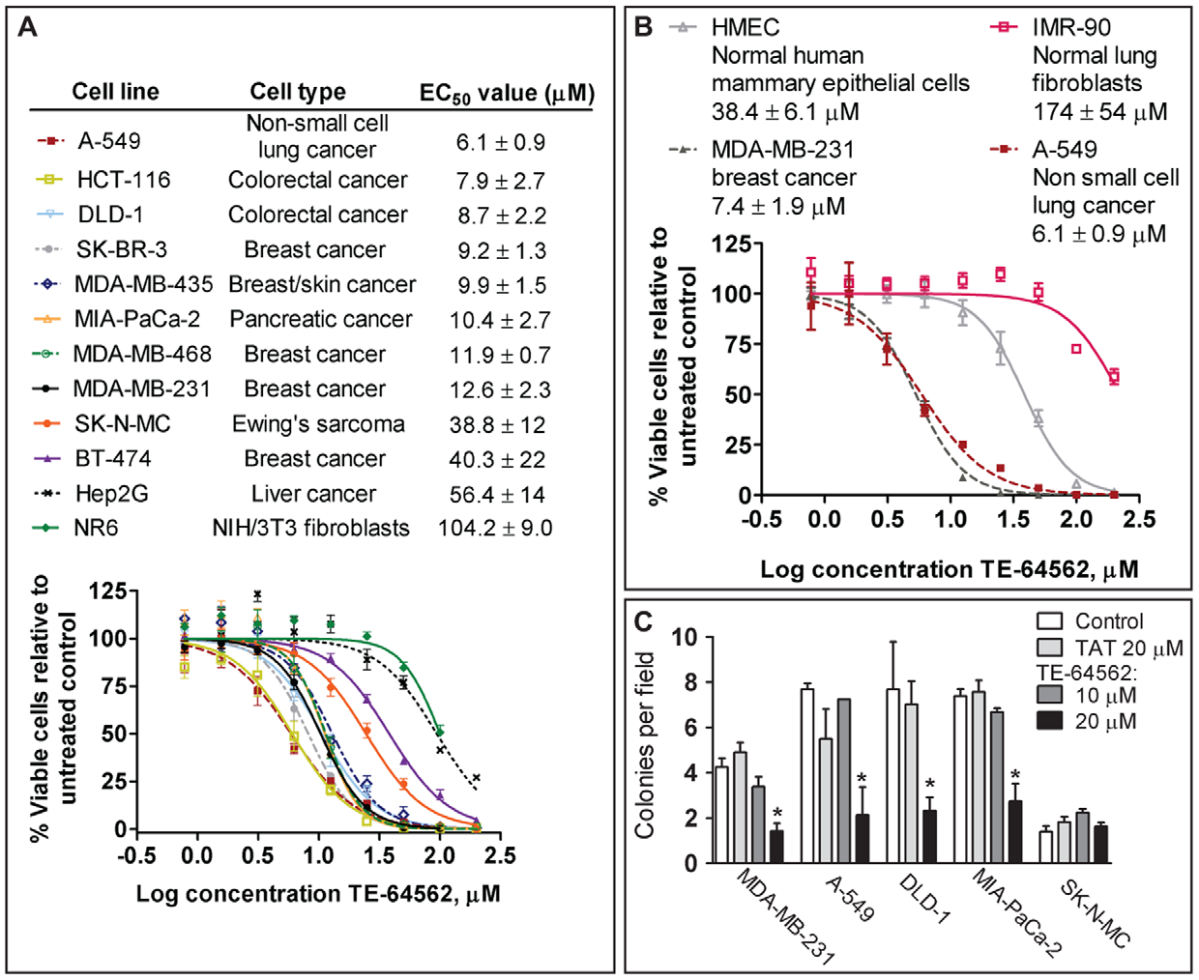
Effect of Tat-645-662 on cell viability and colony growth of various human cancer and normal cell lines. (**A–B**) The indicated cell line was plated overnight, then serum starved overnight and treated with TE-64562 for 24 hours. For the HMEC and MDA-MB-231 cells were treated in HMEC media. Representative dose response curves are shown from one experiment run in triplicate with error bars representing the standard error of the mean. In the legend, the mean EC_50_ values (± standard deviation from two to three independent experiments) derived from the nonlinear fit of dose response curves are shown (generated and fitted in Prism 5.0 GraphPad Software, Inc., USA). Also see Figure S2. (**C**) MDA-MB-231 (breast), A-549 (non-small cell lung), DLD-1 (colo-rectal) MIA-PaCa-2 (pancreatic) and SK-N-MC (neuroepithelioma, EGFR-null) cells were grown in soft agar containing 5% serum alone or treated with Tat peptide (20 µM) or Tat-645-62 peptide (10 or 20 µM) and allowed to grow for 2 weeks with addition of medium or medium containing peptide every 2 days. Means of the counts of four or more plates from at least two independent experiments are plotted. The significantly difference between the mean counts (*P<0.04) for TE-64562 treatment was assessed by comparison to untreated control.

In order to assess whether the activity of TE-64562 varied according to cancer/tissue type and ErbB levels, the cell viability assay was performed on a panel of cancer cell lines. The EC_50_ value of the peptide ranged from 6 to 56 µM, depending on the cancer cell type, relative ErbB levels or the presence of serum (Fig. 2A and S2A).

The cell lines which respond to TE-64562 treatment in the cell viability assay (EC_50_ value of 6.1 to 12.6 µM), have medium to high expression of EGFR and/or ErbB2 [25], [27]. Two cancer cell lines which were more resistant to TE-64562 treatment (EC_50_ value>30 µM) expressed high ErbB3 (BT-474, Hep-G2). Specifically, the breast cancer line BT-474 (EC_50_ 56.4±13.8 µM, Fig. 2A) expresses high levels of ErbB3 and ErbB2 and exhibits ligand-independent ErbB3 activation [25], [28]. The hepatocarcinoma line Hep-G2 (EC_50_ 40.3±22.2 µM, Fig. 2A) expresses a high level of ErbB3 [29]. We confirmed the ErbB expression levels reported in the literature for the resistant cell lines. The ErbB expression levels are plotted relative to expression in MDA-MB-231 cells (Fig. S2B).

Two cell lines were tested which lack EGFR expression. The Ewing sarcoma SK-N-MC line is not an EGFR driven cancer since it lacks EGFR expression (Fig. S2B) [10]. It also lacks ErbB3 expression, but has relatively low ErbB2 expression and some ErbB4 expression (Fig. S2B) [10], [30], [31]. The SK-N-MC cell line was fairly resistant to TE-64562 treatment (EC_50_ 32.7±3.5 µM, Fig. 2A). An example of another EGFR-null cell line with no response to TE-64562 treatment is the NR6 cell line, which displayed an EC_50_ value 104.2±9.0 µM. NR6 cells are an EGFR null clone of NIH/3T3 fibroblasts, which do not express any ErbB2, ErbB3 or ErbB4 [32], [33]. The FAM-conjugated TE-64562 peptide entered SK-NM-C and NR6 cells within approximately 15 minutes of peptide addition (Fig. S1D and S1E), hence the lack of effect is not due to cell impermeability.

In order to test for specificity of TE-64562 for cancer tissue over normal tissue, the activity of TE-64562 was tested in several non-cancerous breast lines (human mammary epithelial cell, HMEC) and compared to the EC_50_ in MDA-MB-231 cells in HMEC media. The peptide showed an EC_50_ value of 38.4±6.1 µM for the HMEC line compared with 7.4±1.9 µM in MDA-MB-231 breast cancer cells (Fig. 2B). The HMEC media contains growth factors and other nutrients that serum-free media lacks, this may cause the EC_50_ of TE-64562 in MDA-MB-231 in HMEC media (7.4±1.9 µM, see Material and Methods for HMEC media formulation) to differ from the EC_50_ in serum-free media (12.6±2.3 µM). Similarly, normal lung fibroblasts (IMR-90) were very resistant to TE-64562 treatment (EC_50_ 174±54 µM) compared to TE-64562 activity in non-small lung cancer cells (A-549, EC_50_ 6.1±0.9) (Fig. 2B). Notably, the IMR-90 line expressed EGFR (Fig. S2B). The decrease in activity of TE-64562 in normal breast and lung cells (HMEC and IMR-90, respectively) compared to breast and lung cancer cells (MDA-MB-231 and A-549, respectively) is indicative of relative selective effects in cancer cells as compared to normal cells.

### The TE-64562 Peptide Inhibited Colony Formation in Soft Agar

In order to determine the effect of the TE-64562 peptide on 3-dimensional cell growth, colony formation in soft agar in the presence or absence of TE-64562 was examined in several cell lines. We chose to test cell lines from different tissues and the ErbB-independent SK-N-MC cell line as a negative control. Colony formation of MDA-MB-231 (breast), A-549 (lung), DLD-1 (colorectal) and MIA-PaCa-2 (pancreatic) cells was reduced by approximately 50% with 20 µM TE-64562 treatment (Fig. 2C). There was not a significant effect on colony growth with 10 µM TE-64562 treatment. TE-64562 treatment had no effect on the formation of SK-N-MC colonies (Fig. 2C).

### The TE-64562 Peptide Induces Non-apoptotic Cell Death After Several Hours and Apoptosis with Overnight Treatment in MDA-MB-231 Cells

**Figure 3:**
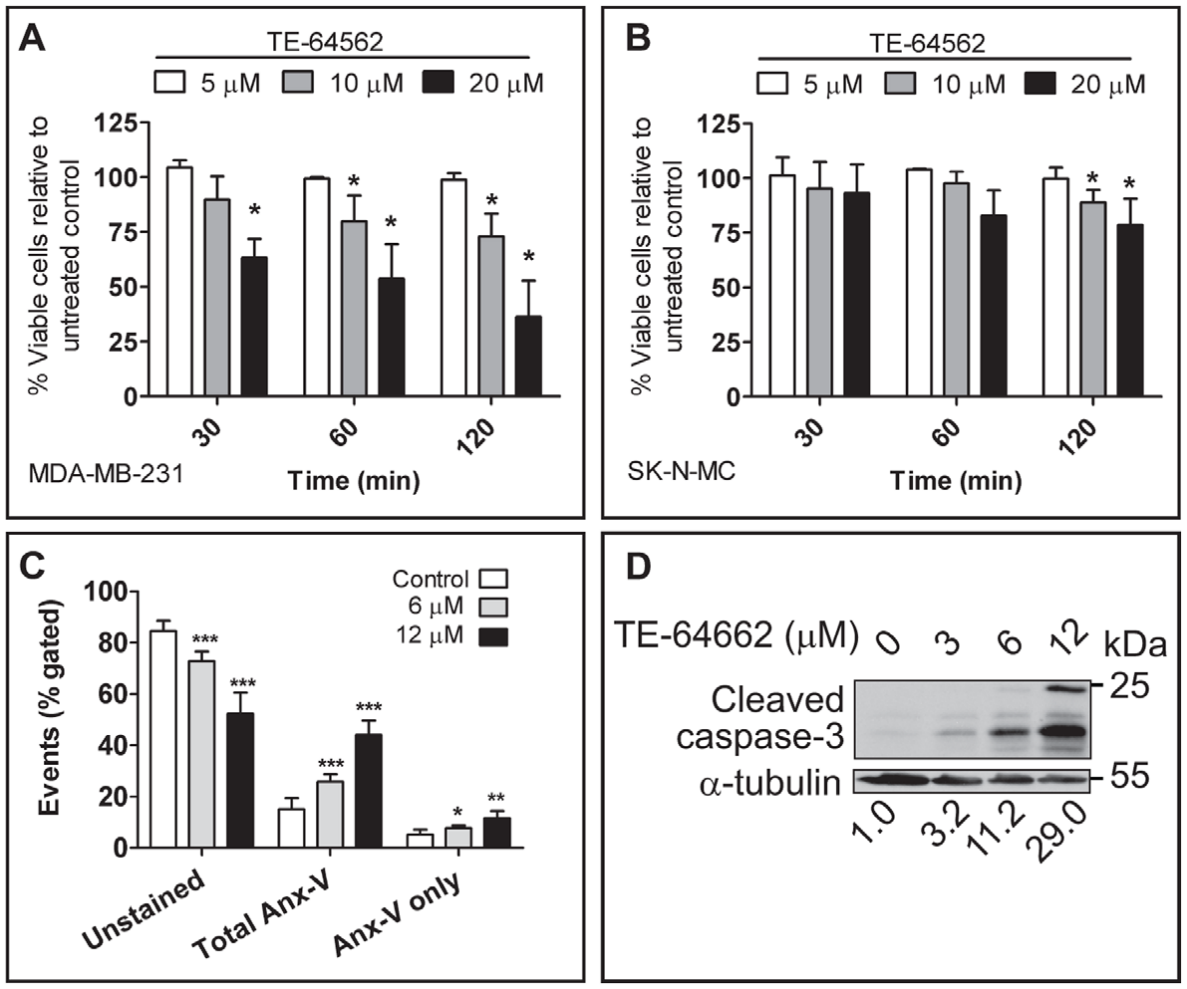
TE-64562 treatment causes early non-apoptotic cell death and induces apoptosis after longer treatment. Serum starved (**A**) MDA-MB-231 cells or (**B**) SK-N-MC cells were treated overnight with 5, 10 or 20 µM of TE-64562 for 0.5, 1 or 3 hours and assayed for cell viability. Data were averaged from three independent experiments and plotted with the error bars representing the standard deviation from the mean. Significant differences were assessed between each treatment condition and untreated control (*P<0.05 from a two-tailed unpaired t test). (**C**) MDA-MB-231 breast cancer cells were serum starved overnight then treated with 0 (control), 6 or 12 µM TE-64562 for 18 hours. Cells were stained with Annexin-V and propidium iodide (PI) and the mean results from four independent experiments are plotted. Unstained cells represent viable cells; total Annexin V staining (Anx-V) is the sum of the Anx-V-stained and dually stained cells which are undergoing apoptosis or are fully apoptotic or necrotic; and Anx-V only stained cells are undergoing apoptosis. Significant differences were assessed between each treatment condition and untreated control (*P = 0.028; **P = 0.008; ***P = 0.004). (**D**) Serum starved MDA-MB-231 cells were treated with 0 (control), 3, 6 or 12 µM TE-64562 for 18 hours. Cells were lysed, analyzed by Western blot and probed for the presence of cleaved caspase-3. Numbers below the blot images represent the quantifications of each band normalized to the respective control. The blot is representative of two independent experiments. All error bars represent standard error of the mean. Also see Figure S3.

We observed that short-term treatment (30 minutes) of MDA-MB-231 cells with TE-64562 caused a visible, morphological change at concentrations ≥10 µM (Fig. S3A). To determine whether the observed effects correlated with a change in cell viability, MDA-MB-231 cells were assayed after 0.5, 1, 3 and 24 hours treatment with TE-64562. There was a significant, dose-dependent reduction in cell viability at the 0.5, 1 and 3 hour timepoints, which does not change from 0.5 to 3 hours treatment (Fig. 3A), but further decreases (by ∼25%) after 24 hours treatment (comparison to EC_50_ value, Fig. 2A). This short-term reduction in cell viability was greatly diminished in the ErbB-independent SK-N-MC cell line (Fig. 3B), indicating that the presence of EGFR is necessary for the early effect on cell viability.

In order to assess whether the reduction in viability caused by TE-64562 after overnight treatment was due to apoptotic cell death, MDA-MB-231 cells were treated and stained with FITC-Annexin V and propidium iodide (PI). Annexin V staining (Fig. 3C and S3B) and caspase-3 activation (Fig. 3D) were both increased in a dose-dependent manner. Compared to control, Annexin V staining increased 1.7- or 2.4-fold on average with a 6 or 12 µM dose of TE-64562, respectively. The total Annexin V staining (including dual staining with PI) increased 1.9- and 3.2-fold on average, with 6 or 12 µM treatment with TE-64562, respectively (Fig. 3C). These results indicate that with 24 hours treatment, TE-64562 induces apoptosis.

### The TE-64562 Peptide Stalls MDA-MB-231 Xenograft Tumor Growth in Nude Mice

**Figure 4:**
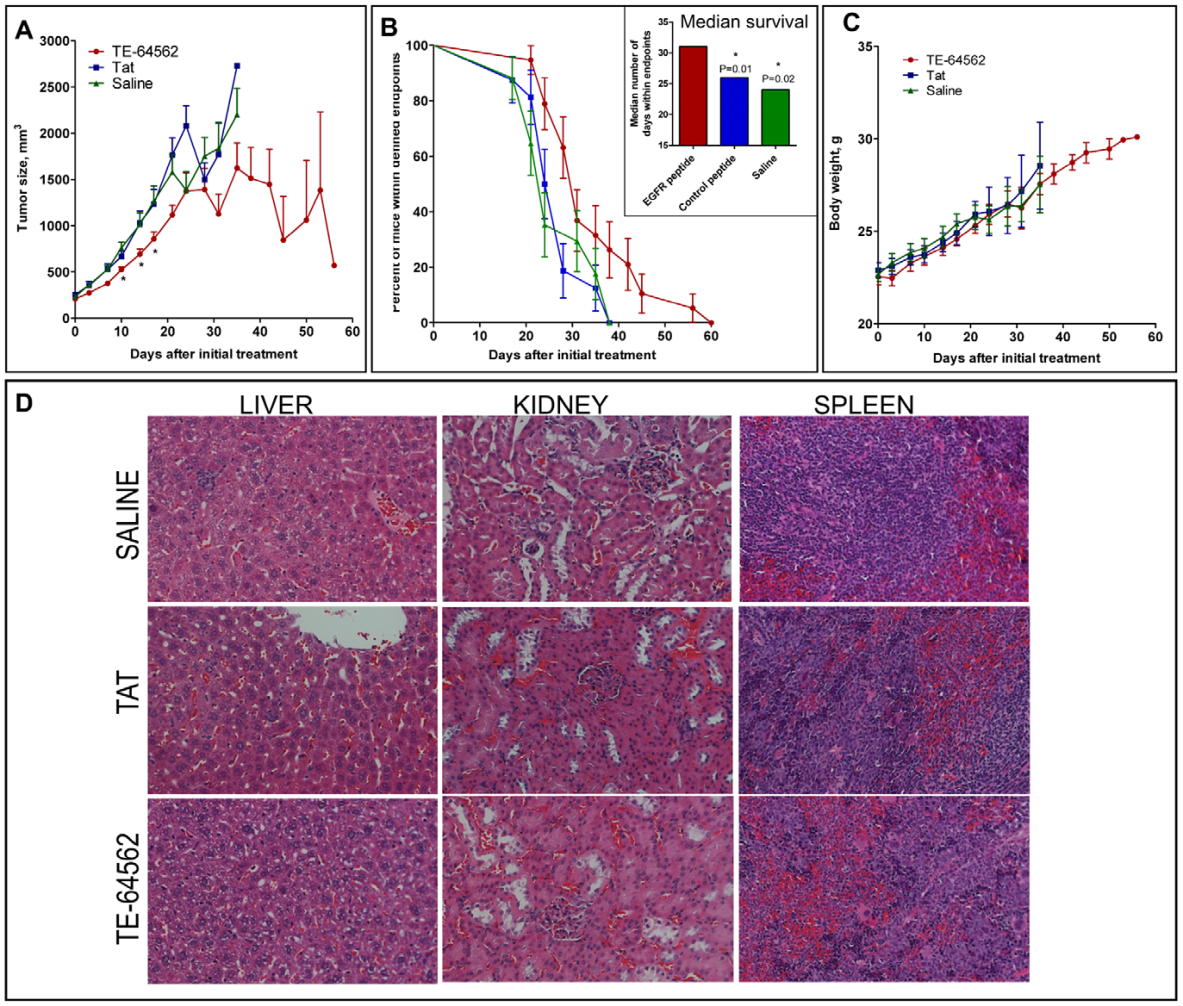
TE-64562 inhibits tumor growth in MDA-MB-231 xenograft tumors and increases survival with no observed toxicity. (**A–C**) MDA-MB-231 xenograft tumors were grown in the subcutaneous flank region of nude mice which were treated bi-weekly with the TE-64562 peptide (40 mg/kg; 7 µmol/kg), Tat-peptide (20 mg/kg; 7 µmol/kg) or vehicle (saline), intraperitoneally. (**A**) The mean tumor size (± standard error of the mean) is plotted over time. The asterisks (*P≤0.0325) indicate that the mean size of the TE-64562 treated tumors is statistically different from the saline- and Tat-treated tumor sizes at that time point. (**B**) The number of mice within endpoints, as defined by tumor size cutoff, tumor ulceration and body conditioning scoring, at each time point are plotted as a Kaplan and Meier survival curve. (**B**, inset) The median survival, the number of days at which the fraction of mice within endpoints is equal to 50%, is plotted for each treatment group. The survival curves for the Tat and Saline groups were compared to the survival curve for the TE-64562 group and the P value was derived using the log-rank (Mantel-Cox) test. The asterisks (*) designate a significant difference with the indicated P values. (**C**) The mean body weight (± standard error of the mean) for each treatment group is plotted over time. (**D**) After 35 days of dosing, organs were collected and fixed. Representative H&E stained sections from liver, kidney and spleen are shown for each treatment group. Results are representative of two independent studies. Also see Figure S4.

In order to evaluate whether the anti-cancer properties of TE-64562 were translatable to anti-tumor activity *in vivo*, MDA-MB-231 xenograft tumors were grown in the subcutaneous flank region of nude mice which were treated bi-weekly with the TE-64562 peptide Tat peptide or vehicle. The MDA-MB-231 cell line was chosen because there was a robust response to TE-64562 in reduction of cell viability and it is tumorigenic. TE-64562 treatment was administered intraperitoneally at 40 mg/kg (7 µmol/kg) and compared to treatment with a molar equivalent amount of the Tat peptide (20 mg/kg, 7 µmol/kg) or vehicle (saline). On average, tumor growth trend was slowed by 15–20% relative to controls 10 to 17 days after treatment initiation and several tumors regressed after 4 weeks of treatment (Fig. 4A and S4A). The TE-64562 treated tumors had notably, but not statistically significant, more dead tissue compared to controls (Fig. S4C). As represented in the Kaplan-Meier survival plot (Fig. 4B), mice treated with TE-64562 survived significantly longer than Tat-treated or vehicle-treated control mice, according to the endpoints defined by tumor-size cutoff and body conditioning scoring. The median survival of TE-64562-treated mice was significantly longer (31 days, log-rank Mantel-Cox test P≤0.023) than the median survival of Tat- and saline-treated mice (26 and 24 days, respectively, Fig. 4B, inset). Similar results were found in a separate study with the same treatment regiment with subcutaneous administration, proximal to the tumor (Fig. S4B).

Toxicity was assessed by monitoring body weight of the mice over the course of the study and histological analysis of organs at the end of 5 weeks of treatment. No significant difference in body weight between the three groups was observed (Fig. 4C). No differences between the treatment groups were observed upon histological examination of post-treatment liver, spleen and kidney samples (Fig. 4D). Hence, although the early cell death is observed in experiments *in vitro*, TE-64562 does not show any significant non-selective toxicity *in vivo*.

### The TE-64562 Peptide Binds to EGFR and Inhibits Dimerization

**Figure 5:**
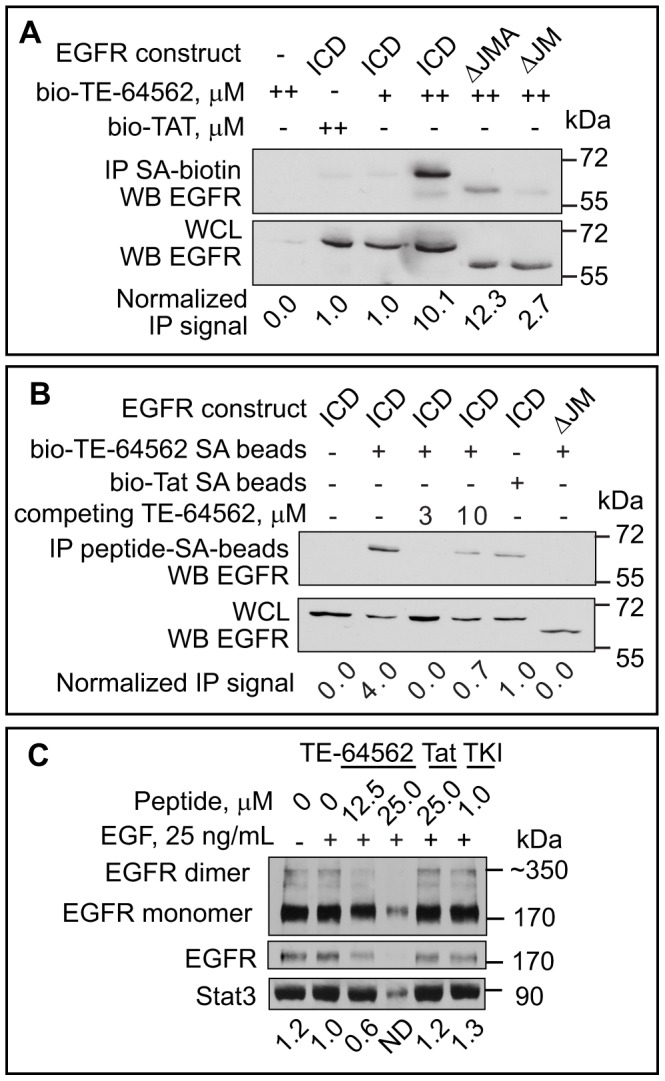
TE-64562 interacts with EGFR and inhibits dimerization. (**A**) SK-N-MC cells were transfected with the intracellular domain (ICD) of EGFR (645–1186) or the ICD of EGFR lacking the entire JXM region (ΔJM) or the JMA region (ΔJMA). Biotinylated peptides at a concentration of 0.1 µM (+) or 0.5 µM (++) were incubated with SK-N-MC cells for 2 hours and precipitated from cellular lysates with streptavidin-coated beads. The resulting bead-precipitates were analyzed by Western blot for the presence of the EGFR constructs. Results are representative of three independent experiments. (**B**) Streptavidin beads were pre-bound with biotinylated peptides and incubated with transfected SK-N-MC lysates. The non-biotinylated version of TE-64562 was added to compete for binding in lanes 3 and 4. The resulting bead-precipitates and lysates were analyzed by Western blot for the presence of the EGFR constructs. Results are representative of two independent experiments. (**C**) Serum starved MDA-MB-231 cells were treated with TE-64562 (12.5 and 25.0 µM), an EGFR specific tyrosine kinase inhibitor (TKI, 1.0 µM), Tat (25.0 µM) or vehicle for 30 minutes, followed by EGF treatment (25 ng/mL) for 10 minutes. Cellular proteins were cross-linked using bis(sulfosuccinimidyl) suberate (BS3), cells were lysed and lysates analyzed by Western blot for EGFR. The quantification of the dimer band is shown. The EGFR dimer band 25.0 µM TE-64562 was not detectable (N.D.). Results are representative of three independent experiments.

To test whether the cellular activity of TE-64562 was driven by an interaction with EGFR, a binding assay was performed using biotinylated peptides and streptavidin beads in SK-N-MC cells (exhibit no endogenous EGFR expression) transfected with various EGFR constructs. We hypothesized that if the TE-64562 peptide mimics the structural role of the EGFR JMA domain, then the peptide would bind to EGFR at the JXM region. To test whether the JXM region was essential for binding, cells were transfected with the intracellular domain (ICD) of EGFR (645–1186), the ICD of EGFR lacking the JMA domain (ΔJMA) or the ICD of EGFR lacking the entire JXM (ΔJM) region. The biotinylated TE-64562 peptide bound to the ICD of EGFR at 0.5 µM (Fig. 5A, lane 4) but not at 0.1 µM (Fig. 5A, lane 3), whereas the biotinylated Tat peptide did not show any binding (Fig. 5A, lane 2). The binding was reduced when the JMA domain (ΔJMA) or the entire JXM domain (ΔJM) was lacking (Fig. 5A, lanes 5 and 6, respectively), indicating that the region of EGFR that TE-64562 binds is within the JXM domain.

In a reverse experiment, the biotinylated peptides were attached to streptavidin beads and incubated with SK-N-MC lysates, expressing the ICD or ΔJM constructs. The TE-64562 peptide bound to the ICD of EGFR (Fig. 2B, lane 2) and not the EGFR construct lacking the JXM domain (ΔJM; Fig. 5B, lane 6). The non-biotinylated version of TE-64562 was incubated with the bead-lysate mixture to compete for the binding of the biotinylated peptide. The binding of EGFR ICD to the peptide-conjugated beads was diminished with 3 and 10 µM competing peptide (Fig. 5B, lanes 3 and 4, respectively). The small amount of EGFR bound with 10 µM of the competing, non-biotinylated peptide was most likely due to oligomerization of the free peptide with the streptavidin-bound peptide, which baits EGFR. The Tat peptide bound weakly to the EGFR ICD (Fig. 5B, lane 5). Overall, these results indicate that TE-64562 reversibly binds to EGFR at the JXM domain.

In order to test whether treatment with TE-64562 effects dimerization of EGFR, MDA-MB-231 cells were treated with increasing amounts of TE-64562, Tat or TKI for 30 minutes followed by EGF. Proteins were cross-linked and analyzed by Western blot for the presence of an EGFR dimer band. Dimerization of EGFR was decreased by TE-64562 treatment at 12.5 µM (Fig. 5C). Treatment with 25 µM TE-64562 was fairly toxic to the cells and caused a reduction in the loading control (Stat3), indicating a substantial effect on cell viability. Although, the level of total EGFR is affected by TE-64562 treatment, the dimer:monomer ratio is also decreased with TE-64562 treatment.

### TE-64562 Reduces Total and Phospho-EGFR Levels and Prolongs EGFR Phosphorylation

**Figure 6:**
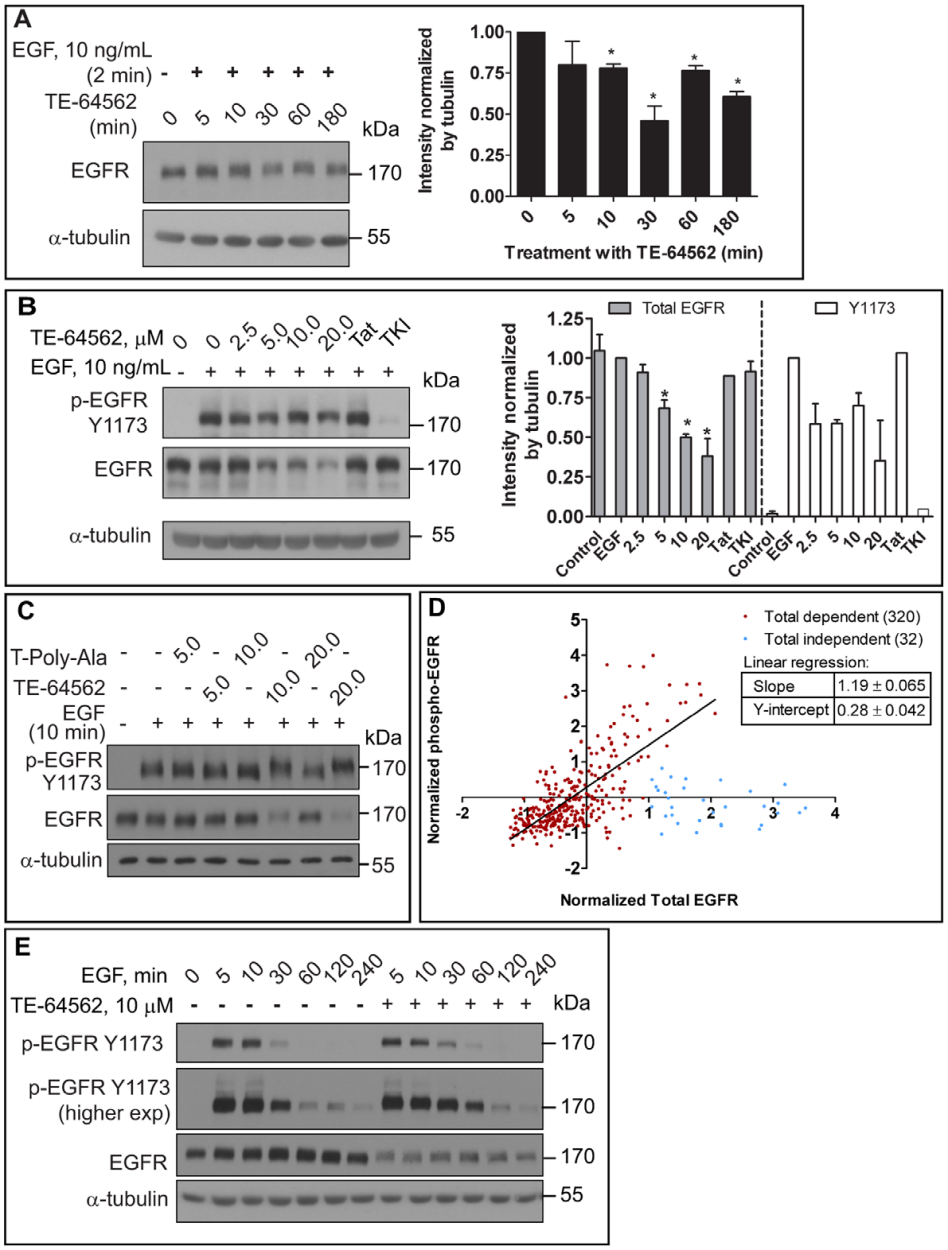
TE-64562 causes EGFR down-regulation and prolongs EGFR phosphorylation. (**A**) Serum starved MDA-MB-231 cells were treated with EGF for 2 minutes followed by TE-64562 (10 µM) treatment for the indicated amounts of time. The intensity of the EGFR bands was quantified with respect to the intensity of the respective α-tubulin bands. Mean intensity values are plotted from two independent experiments and error bars represent the standard error of the mean. Significance was assessed by a two-tailed, unpaired t test (*P<0.026). (**B**) Serum starved MDA-MB-231 cells were treated with TE-64562 (2.5, 5.0, 10.0 and 20.0 µM), Tat (20 µM), an EGFR specific tyrosine kinase inhibitor (TKI, 2.0 µM) or vehicle for 30 minutes, followed by EGF treatment (10 ng/mL) for 10 minutes. Results are representative of three independent experiments. The intensity of the EGFR (N = 3 to 7, depending on concentration) or phospho-EGFR (N = 2) bands were quantified with respect to the intensity of the respective α-tubulin band. Mean intensity values are plotted from two (phospho) or three or more (total) independent experiments and error bars represent the standard error of the mean. Significant differences (Mann-Whitney test) were assessed between each treatment condition and untreated control (*P<0.03). (**C**) Serum starved MDA-MB-231 cells were treated with TE-64562 (5.0, 10.0 and 20.0 µM) or T-Poly-Ala control peptide (5.0, 10.0 and 20.0 µM) or vehicle for 30 minutes, followed by EGF treatment (10 ng/mL) for 10 minutes. Results represent one of three independent experiments. (**D**) Correlation plot of breast cancer data from The Cancer Genome Atlas. The normalized EGFR protein expression data from antibody array data was plotted for each individual in the study. Shown in red are the 320 individuals who showed a positive correlation between EGFR expression and phospho-EGFR Y1173 levels. Shown in blue are the 32 individuals who did not show a correlation. Plotting and linear regression were performed in Prism 5.0 (GraphPad Software, Inc., USA). (**E**) Serum starved MDA-MB-231 cells were treated with TE-64562 (10 µM) or vehicle for 30 minutes followed by EGF (10 ng/mL) for 5, 10, 30, 60 120 or 240 minutes. Cell lysates were collected and analyzed by Western blot for phospho-EGFR (Y1173) and EGFR. Blots were stripped and re-probed for α-tubulin. Also see Figure S5.

In order to test whether the peptide has an effect on EGFR levels, MDA-MB-231 cells were treated with EGF for two minutes followed by treatment with 10 µM TE-64562 for 5, 10, 30, 60 and 180 minutes, then analyzed for the presence of EGFR. By 30 minutes, EGFR levels were significantly decreased by almost 50% compared to untreated control (Fig. 6A) and the EGFR remained diminished for up to 3 hours. In order to test whether the peptide has a dose-dependent effect on EGFR levels even without ligand occupancy, MDA-MB-231 cells were treated with increasing concentrations of TE-64562 for 30 minutes, followed by EGF treatment for 10 minutes and analyzed for the presence of EGFR. At TE-64562 concentrations of 5 µM and higher, a significant reduction in EGFR levels was observed (Fig. 6B).

In order to test whether the peptide has a dose-dependent effect on EGFR phosphorylation levels, MDA-MB-231 cells were treated with increasing concentrations of TE-64562 for 30 minutes, followed by EGF treatment for 10 minutes and analyzed for the presence of phospho-EGFR at Y1173, a known auto-phosphorylation site. Using total EGFR levels as the baseline, the phosphorylation of EGFR at Y1173 is unaffected by the presence of TE-64562 (Fig. 6B). However, when normalized to α-tubulin, there is a decrease in the level of Y1173 phosphorylated EGFR (Fig. 6B). Other EGFR phosphorylation sites (Y992, Y1045, Y1068 and Y1086) were affected similarly by TE-64562 treatment (Fig. S5). This is reflective of a decrease in the levels of phosphorylated EGFR upon TE-64562 treatment. However, as total levels of EGFR also decrease, it is not reflective of inhibition of kinase activity. We have previously observed a similar phenomenon (in reverse) when levels of phospho-CaMKII increase as levels of total CaMKII increase due to acute translation during synaptic plasticity [34]. To test the possibility that the effects on EGFR were due to the positively charged nature of TE-64562, the effect of the T-Poly-Ala peptide (Table 1) on EGFR phosphorylation and levels was tested. The T-Poly-Ala peptide did not show any effect on EGFR phosphorylation or total EGFR levels (Fig. 6C).

As an indication of whether this phenomenon of simultaneously reducing total and phospho-levels is relevant for therapy, we looked for a correlation between phosphorylated and total EGFR levels in patient data in The Cancer Genome Atlas (TCGA). We hypothesized that if there is a positive correlation between phospho-EGFR and its total level, then effectively reducing both forms of the receptor should be as therapeutically effective as or more effective than inhibiting kinase activity. As shown in Figure 6D, there is a linear relationship between the total and phospho-EGFR across a majority (320) of patient samples and no relationship with a minor subset (32) of patient samples, where EGFR was expressed at higher than normal levels but phospho-EGFR levels were unchanged.

Although TE-64562 did not change EGFR kinase activity at a single timepoint, the effect TE-64562 treatment EGFR phosphorylation was tested as a function of time. MDA-MB-231 cells were pre-treated with TE-64562 for 30 minutes, followed by EGF treatment for increasing amounts of time (Fig. 6E). It was observed that EGFR remained phosphorylated at 60 minutes EGF treatment in the presence of TE-64562; whereas, without TE-64562 pre-treatment, the phosphorylation of EGFR at 60 minutes was reduced to nearly basal level.

### TE-64562 Modulates Multiple EGFR Signaling Pathways

**Figure 7:**
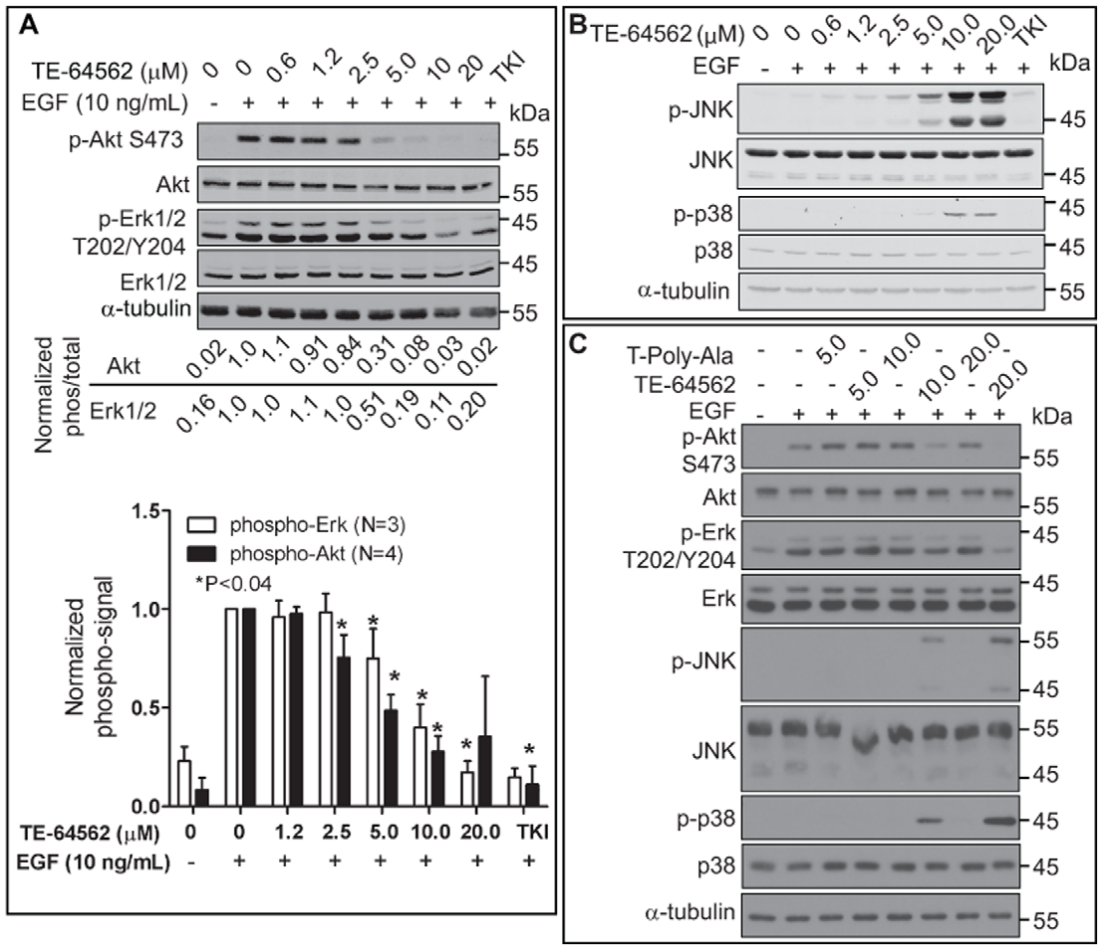
TE-64562 effects downstream EGFR signaling. (**A**) Serum starved MDA-MB-231 cells were treated with TE-64562 (0.6, 1.2, 2.5 5.0 10 and 20 µM), an EGFR specific tyrosine kinase inhibitor (2.0 µM, TKI) or vehicle for 30 minutes, followed by EGF treatment (10 ng/mL) for 10 minutes. Cell lysates were collected and analyzed by Western blot for (**A**) phospho-Akt (S273), total Akt, phospho-Erk (p44/p42, T202/Y204) and total Erk. Blots were stripped and re-probed for α-tubulin. The Erk (N = 3) and Akt (N = 4) data from separate experiments, all of which are shown in Figure S6, were analyzed. The normalized (ratio of phospho to total intensity) data were plotted as mean values from with the error bars representing the standard error of the mean. The significant differences (*P<0.04) were assessed between each treatment condition and the EGF-treated control (lane 2). (**B**) Serum starved MDA-MB-231 cells were treated with TE-64562 (0.6, 1.2, 2.5 5.0 10 and 20 µM), an EGFR specific tyrosine kinase inhibitor (2.0 µM, TKI), Tat (20 µM) or vehicle for 30 minutes, followed by EGF treatment (10 ng/mL) for 10 minutes. Results represent one of three independent experiments. Also see Fig. S6. (**C**) Serum starved MDA-MB-231 cells were treated with TE-64562 (5.0, 10.0 and 20.0 µM) or T-Poly-Ala control peptide (5.0, 10.0 and 20.0 µM) or vehicle for 30 minutes, followed by EGF treatment (10 ng/mL) for 10 minutes. Results are representative of three independent experiments.

Treatment with TE-64562 did not reduce EGFR phosphorylation but prolonged it, downregulated total EGFR levels and inhibited EGFR dimerization. We hypothesized that the outcome of these effects may result in changes in downstream EGFR signaling. To assess this, Akt and MAPK signaling were examined in MDA-MB-231 cells. Akt and Erk phosphorylation were inhibited in a dose dependent manner (Fig. 7A and S6) and in MIA-PaCa-2 cells (Fig. S6F) treated with TE-64562. Erk phosphorylation significantly decreased with 10 and 20 µM TE-64562 treatment (Fig. 7A). Akt phosphorylation significantly decreased with 2.5, 5, 10 and 20 µM TE-64562 treatment (Fig. 7A). To ensure that the effect was not due to the positively charged nature of TE-64562, the effect of the T-Poly-Ala peptide on Erk and Akt phosphorylation was tested. Treatment with the T-Poly-Ala peptide did not show any effect on p-Erk or p-Akt levels, at concentrations where TE-64562 reduced Erk and Akt phosphorylation (Fig. 7C). From these results, we conclude that treatment with the TE-64562 peptide inhibits downstream EGFR signaling at Akt and Erk.

Since TE-64562 affected Erk signaling, we assessed whether there was an effect on any other MAPK signaling pathways by examining JNK and p38 signaling. The dose response data showed that TE-64562 induced JNK and p38 phosphorylation maximally at 10 and 20 µM, in the presence of EGF, in MDA-MB-231 cells (Fig. 7B) and MIA-PaCa-2 cells (Fig. S3F). Since activation of p38 and JNK is associated with stress signaling [35], the results indicate that TE-64562 may induce some cellular stress leading to cell death. This effect is specific to TE-64562, as the T-Poly-Ala control peptide did not stimulate JNK or p38 phosphorylation (Fig. 7C).

### TE-64562 Treatment Inhibits Akt and Erk Signaling in MDA-MB-231 Xenograft Tumors

**Figure 8:**
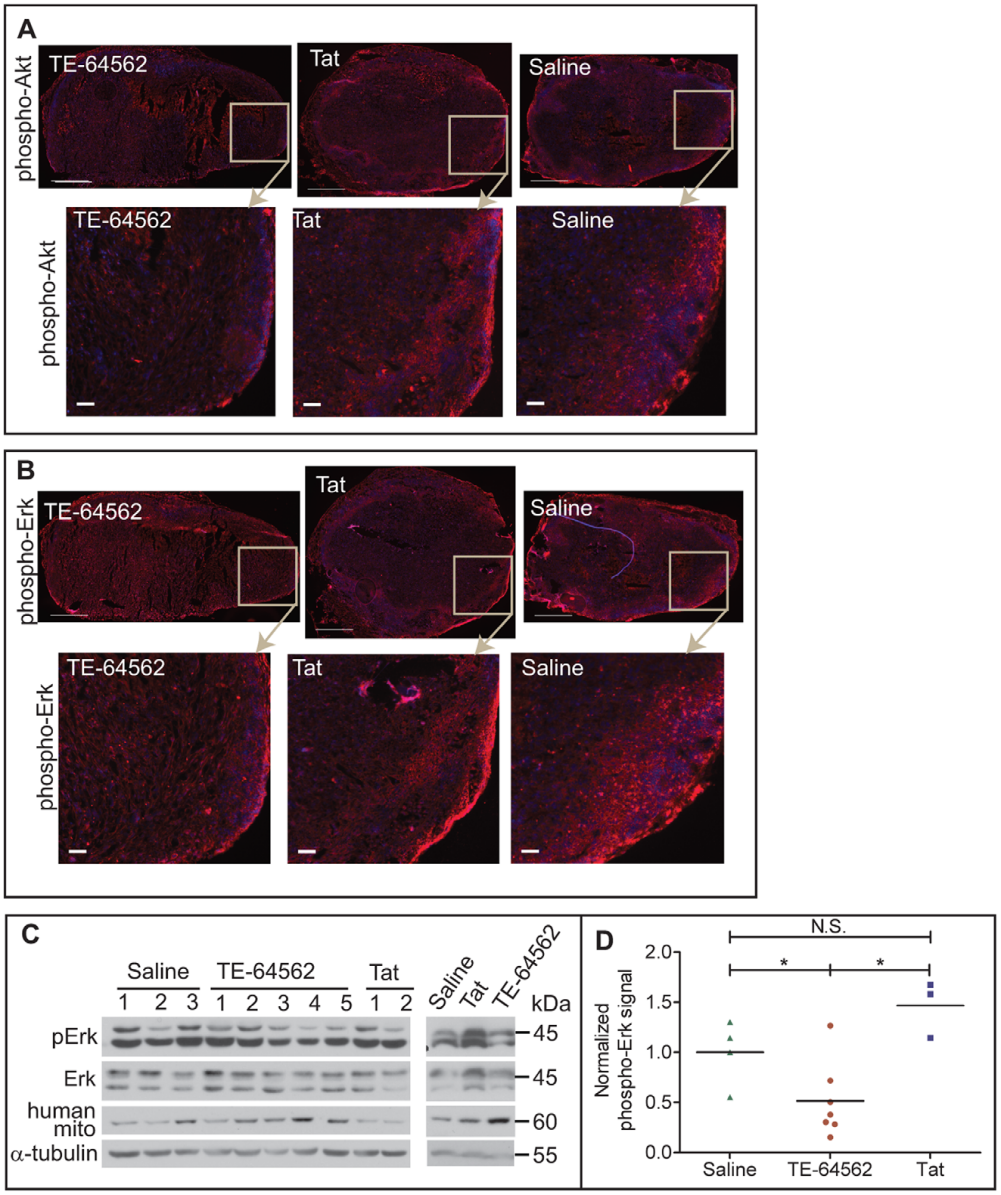
TE-64562 treatment reduces Akt and Erk phosphorylation in MDA-MB-231 xenograft tumor tissue. (**A–B**) Nude mice bearing subcutaneous, MDA-MB-231 xenographic tumors were injected with the TE-64562 peptide (40 mg/kg; 7 µmol/kg), Tat-peptide (20 mg/kg; 7 µmol/kg) or vehicle (saline), intraperitoneally for four days, once per day. On the last day, the mice were injected 30 minutes prior to extracting the tumor. Frozen tumor sections were stained for (**A**) phospho-Akt (S473) or (**B**) phospho-Erk and counterstained with DAPI. Representative stained tumor sections are shown with the area in the box enlarged in the images below each section. Large scale bars = 500 µm and small scale bars = 50 µm. (**C**) A ∼1–2 mm cross-sectional slice of the tumor was lysed in RIPA buffer by sonication and the resulting lysates were analyzed by Western blot. Each lane represents a tumor from a different mouse. (**D**) Western blot data is quantified and plotted. Each treatment group was compared statistically (*P≤0.0364). Error bars represent the standard error of the mean. Also see Figure S6.

MDA-MB-231 tumors in nude mice were allowed to grow to approximately 60 to 100 mm^3^ and mice were injected intraperitoneally with TE-64562 (40 mg/kg), Tat (20 mg/kg) or saline for 5 days. Tumors were removed 30 minutes after the last injection and analyzed. Frozen tumor sections were stained for phospho-Akt and phospho-Erk and a representative tumor section from each treatment group was imaged (Fig. 8A and 8B, respectively). The phospho-Akt and phospho-Erk staining is diminished in the TE-64562-treated tumors relative to Tat- and saline-treated control tumors. A cross-sectional tumor section was lysed and analyzed by Western blot for phospho-Erk. In five out of six mice, the phospho-Erk level was inhibited by TE-64562 treatment (Fig. 8C). Mouse tissue and blood showed a high amount of total Erk (p44) and a low amount of basal phospho-Erk. In order to compare the level of phospho-Erk to the human tissue, the phospho-signal was normalized to a human tissue marker (human mitochondria). Quantification of the Western blot data showed that phospho-Erk was significantly reduced in TE-64562-treated tumors compared to Tat- and saline-treated control tumors (Fig. 8D).

## Discussion

Recent studies have established the critical role of the JXM domain in the fully active conformation of EGFR [10], [11], [12], [13], [18]. This evidence lead to our hypothesis that a peptide derived from the JMA or JMB region would interfere with EGFR activity by mimicking its respective role in the full-length protein (latch formation or anti-parallel helical dimer). Through testing of various peptides, it was observed that Tat-conjugation was necessary for cell permeability of the JMA-region peptide, but did not allow for cell permeability of the JMB-region peptide (Fig. 1 and S1). Moreover, TE-664-682 and Tat did not reduce the viability of MDA-MB-231 cells, while TE-64562 reduced the viability of these cells with an EC_50_ of 12.6 µM. Control peptides, including the T-Poly-Ala peptide which maintains the positively charged amino acids while substituting most amino acids with alanine, were shown to have greatly reduced activity against MDA-MB-231 cell viability. The JMA-region peptide, TE-64562, was further assayed in a panel of human cell lines from different tissues. Most cancer cell lines showed an EC_50_ in the range of 6 to 13 µM (Fig. 2A) and expressed some level of EGFR (Fig. S2B) [25], [27]. The cell lines that had significantly higher EC_50_ values expressed either low EGFR with high ErbB3 (BT-474 and Hep-G2); no EGFR, low ErbB2 and high ErbB4 (SK-N-MC); no ErbB family expression (NR6) or were non-cancerous (HMEC and IMR-90) (Fig. 2A, 2B and S2B). From these results, we conclude that TE-64562 displayed relative selectivity of activity in cancer cell lines where EGFR is expressed and contributes to proliferation and survival.

TE-64562 displayed activity against growth in soft agar of several cancer cell lines which are EGFR positive (MDA-MB-231, A-549, DLD-1, MIA-PaCa-2) but no activity against growth of the EGFR-null SK-N-MC cell line (Fig. 2C). Furthermore, systemic administration of the TE-64562 peptide reduced growth of MDA-MB-231 tumors in mice and prolonged survival, without any gross toxicity or weight loss (Fig. 4). Taken together these observations indicate that TE-64562 can function as a selective anti-cancer drug for tumors that are EGFR positive.

The mechanism of action of TE-64562 was EGFR-selective, but complex. EGFR binding, EGFR levels, kinetics of phosphorylation and downstream signaling were assayed. It was determined that TE-64562 binds EGFR, inhibits dimerization and causes a down-regulation of EGFR. TE-64562 reduces the level of phosphorylated EGFR with respect to total cellular proteins, using α-tubulin as a surrogate. The peptide does not appear to have an effect on intrinsic kinase activity as the total EGFR levels decrease at a similar rate (Fig. 5 and 6). In order to assess whether the total reduction of EGFR levels (total and phosphorylated) could be a valid therapeutic mechanism, we assessed the protein expression levels of EGFR and phospho-EGFR in patient data from the TCGA. There was a strong correlation between the levels of the phosphorylated and total protein, indicating that reducing both simultaneously could be an effective therapeutic strategy (Fig. 6D). EGF-induced phosphorylation of EGFR was prolonged by 30 minutes with TE-64562 treatment (Fig. 6E). Taken together, these observations suggest that TE-64562 may reduce the unphosphorylated form of the receptor more effectively than the phosphorylated form, allowing for an apparent longer duration of kinase activity. Upon binding the unphosphorylated EGFR, TE-64562 may cause EGFR to assume an unnatural conformation that accelerates its internalization and degradation. Because TE-64562 inhibits Akt and Erk (Fig. 7), we assume that this unnatural EGFR conformation decreases its ability to signal downstream, even though phosphorylated receptor is present. Since EGFR plays a role in cellular stress signaling and EGFR clustering is associated with stress [35], [36], it is possible that the EGFR conformation induced by TE-64562 mimics the stress sensory mode of EGFR thereby activating p38 and JNK. This stress signaling can play a role in the short-term (occurring from 0.5 to 3 hours) non-apoptotic cell death induced by TE64562 treatment, as has been observed in cardiomyocytes [24]. The biochemical mechanism of reducing Erk and Akt activation was shown to be functional in the tumors (Fig. 8). This suggests that the anti-tumorigenic effects involve the inhibitory effects of TE-64562 on downstream EGFR signaling.

In summary, the data indicate that a new approach to target EGFR in cancer is at the juxtamembrane region. The TE-64562 peptide could potentially serve as a therapeutic. Additionally, the peptide could be used as a probe in screens to find small molecules which mimic its effects. Further, we propose that modulating, rather than completely inhibiting enzyme activity or ligand-binding, EGFR activity is promising to overcome the mechanisms of resistance that are encountered by current EGFR therapies.

## Materials and Methods

### Ethics Statement

All animal experiments adhered to a protocol approved by the Institutional Animal Care and Use Committee (IACUC) at the Mount Sinai School of Medicine and were performed according to the Office of Laboratory Animal Welfare (OLAW, National Institutes of Health) and Animal Welfare Act (AWA, United States Department of Agriculture) guidelines.

### Materials

All peptides were purchased from Genscript (Piscataway, NJ). The high performance liquid chromatography reports indicated at least 92% purity and the peptide masses were confirmed by mass spectrometry. Antibodies for phospho-Akt (S473), Akt, phospho-Erk, Erk, phospho-JNK, JNK, phospho-p38, p38 and EGFR were purchased from Cell Signaling Technology (Danvers, MA). The phospho-EGFR Y1173 antibody was purchased from Millipore. The human mitochondria antibody was purchased from Abcam (Cambridge, MA). The EGFR-specific tyrosine kinase inhibitor (TKI; Cyclopropanecarboxylic acid-(3-(6-(3-trifluoromethyl-phenylamino)-pyrimidin-4-ylamino)-phenyl)-amide) was purchased from Calbiochem (USA).

### Cell Lines

The MDA-MB-231, SK-BR-3, MDA-MB-435, MDA-MB-468, BT-474, DLD-1, A-549, MIA-PaCa-2 and SK-N-MC cell lines were obtained from the American Type Culture Collection (ATCC, Manassas, VA) and cultured according to ATCC guidelines. The Hep-G2 and HCT-116 cell lines were generously provided by Dr. Arthur Cederbaum and Dr. Stuart Aaronson, respectively, of the Mount Sinai School of Medicine, NY, were originally from the ATCC and cultured according to ATCC guidelines. The NR6 cells were generously provided by Dr. Alan Wells of the University of Pittsburgh, PA and cultured in MEM-α supplemented with non-essential amino acids, 7.5% FBS and antibiotics (penicillin/streptomycin) [37]. The human mammary epithelial cell (HMEC) lines were established and generously provided by Dr. Martha Stampfer of Lawrence Berkley National Laboratory, CA [38], [39]. As described previously [40], [41], [42], HMEC lines were cultured in 50% mammalian epithelial growth medium (MEGM, Lonza, Allendale, NJ) and 50% DMEM/F12 medium with various supplements at 37°C and 5% CO_2_. MEGM was supplemented with bullet kit (50%, Lonza, Allendale, NJ) containing transferring, isoproterenol and glutamine. DMEM/F12 media was supplemented with insulin, tri-iodothyronine, β-estradiol, hydrocortisone, fetal calf serum, EGF, glutamine and cholera toxin.

### Cell Viability Assay

Cells were plated into a 96-well plate (5,000 to 10,000 cells per well) in full growth media. The following day, media is exchanged for serum starved media (0.1% BSA in growth media without FBS) and incubated overnight. Cells were treated for 24 hours with peptides at varying concentrations range (0.78 to 200 µM). The cell viability is read using luminescent cell viability dye (CellTiter-Glo, Promega Corporation, USA) by adding 20 µL of dye to each well containing 100 µL of treated media. The cell viability is calculated by dividing each luminescent reading by the average of the luminescent readings for control, untreated cells. Assays are run in triplicate. Dose-response curves were generated and fitted in Prism 5.0 (GraphPad Software, Inc., USA). The EC_50_ values were generated using the log inhibitor-normalized response variable slope function (Y = 100/(1+10∧((LogEC_50_-X)*HillSlope)). EC_50_ values are shown with standard deviation values (S.D.) from at least three independent experiments. For comparison of MDA-MB-231 with HMEC, the cells were not serum starved and plated and treated in HMEC media.

### Fluorescent Confocal Microscopy

MDA-MB-231 cells (1×10^5^ cells per plate) were plated on 35 mm glass-bottom plates (MatTek Corporation, Ashland, MA), allowed to adhere overnight, then serum starved overnight and analyzed the following day in HBSS (Hank’s balanced salt solution). For real-time uptake of the FAM-labeled peptides, after recording background images, the FAM-labeled peptides were added and cells were imaged every two minutes over approximately 30 minutes. After 30 minutes, images were taken less frequently. Images from representative timepoints are shown. For overnight treatment, the cells were treated in serum-starved media, exchanged into HBSS the following day and imaged.

### Colony Formation in Soft Agar

Cells (1×10^5^ to 2×10^5^ per plate) were suspended in soft agar containing 5% serum and dosed with vehicle, Tat peptide or the TE-64562 peptide and allowed to grow for 2 to 3 weeks with periodic dosing to keep the dosing media fresh and the agar hydrated. Viable colonies were stained with iodonitrotetrazolium chloride at 0.5 mg/mL overnight. Colonies larger than 0.3 mm in each field were manually scored using a light microscope.

### Apoptosis Assays

MDA-MB-231 cells were plated in 10 cm dishes, grown to 80% confluence and serum starved overnight. The TE-64562 peptide was added to at the indicated concentrations and incubated at 37°C for 18 hours (overnight). Cells were collected by trypsination, washed and suspended in binding buffer and stained according the manufacturer’s protocol (BD Biosciences, Annexin-V-FITC Apoptosis detection kit). Cells were analyzed on a FACscan instrument using BD Biosciences (USA) CellQuest software. For caspase-3 cleavage, treated cells were lysed using RIPA and analyzed by SDS-PAGE followed by Western blotting using an antibody specific for cleaved caspase-3 (Cell Signaling Technology, Danvers, MA).

### MDA-MB-231 Xenografts in Nude Mice

NCR-nude female athymic mice were purchased from Taconic Farms, Inc. Mice were injected in the flank region with 1.5×10^6^ MDA-MB-231 cells, while anesthetized with ketamine and xylazine. Prior to treatment, tumors were measured in three dimensions using a caliper and tumor volume was calculated by multiplying the three measured dimensions by 0.5 (V = 0.5*l*w*h). Once the tumors reached a minimum size of 100 mm^3^, mice were injected intraperitoneally, twice a week, with 200 µL of the TE-64562 peptide (40 mg/kg; 7 µmol/kg), Tat peptide in PBS (20 mg/kg; 7 µmol/kg) or PBS. Endpoints were defined as the tumor size reaching 2000 mm^3^ or 20 mm in any dimension, significant weight loss occurring or if the mouse appearing unhealthy according to body conditioning scoring standards. For histological analysis, organs were collected post-sacrifice and fixed in 4% formaldehyde in PBS, followed by paraffin embedding. Sections (6 µm thick) were mounted onto positively charged slides and H&E stained. Images were captured at 20× resolution on a Zeiss Axioplan 2 microscope.

### EGFR-peptide Biotin-binding Assay

The EGFR constructs (ICD 645–998; ΔJMA 663–998; ΔJM 677–998) were obtained from the laboratory of Dr. Graham Carpenter of Vanderbilt University and described previously [12]. SK-N-MC cells were plated in 10-cm dishes, grown to 90% confluence, transfected with the indicated EGFR construct using Lipofectamine 2000 (Invitrogen, USA) for 6 hours and serum starved overnight. The cells were treated with the biotinylated peptides for 2 hours, washed with phosphate buffer containing 500 mM NaCl pH 7.4, and then lysed by sonication in immunoprecipitation buffer (50 mM Hepes pH 7.4, 150 mM NaCl, 0.1% Tween-20, 10% glycerol, 1 mM EDTA, 1 mM DTT, 0.1 mM PMSF and protease inhibitor cocktail (Halt, Thermo Scientific, USA). The lysates were incubated with pre-washed, streptavidin-coated beads overnight at 4°C with rotation. For the reverse experiment, biotinylated peptides were incubated with streptavidin-coated beads for 2 hours at 4°C with rotation and washed. Transfected SK-N-MC cells were lysed, as above and incubated with the peptide-conjugated beads overnight at 4°C with rotation. The resulting bead-precipitates were washed and analyzed by Western blot with the indicated antibodies following standard procedures and visualized by chemiluminescence.

### Cell Treatment and Lysate Collection for Western Blot Analysis

Cells were plated in 10-cm dishes, grown to 80% confluence, serum starved overnight, treated and analyzed. For dose response assays, the peptides, EGFR-specific tyrosine kinase inhibitor (TKI) or control was added to each plate and incubated at 37°C for 30 minutes, followed by EGF (10 ng/mL) for an additional 10 minutes at 37°C. For timecourses, TE-64562 and/or EGF were added and the cells were incubated at 37°C for the indicated amounts of time. For EGFR dimer detection, cells were cross-linked after treatment using BS^3^ crosslinker (bis[sulfosuccinimidyl] suberate, Thermo scientific, Inc) according to the manufacturer’s instructions. Immediately following treatment, cells were put on ice, washed twice with cold Tris-buffered saline (TBS, pH 7.4) and lysed with radio-immunoprecipitation buffer (50 mM Tris pH 7.5, 100 mM NaCl, 1% NP-40, 0.5% deoxycholate, 0.1% SDS, 1 mM EDTA and protease inhibitor cocktail). After protein concentration determination, cell lysates were analyzed by Western blot analysis with the indicated antibodies following standard procedures and visualized by chemiluminescence. Images were quantified using ImageJ Version 1.43 u.

### Immunofluorescent and Western Blot Analysis of Tumor Tissue

Nude mice bearing subcutaneous, MDA-MB-231 xenographic tumors were injected with the TE-64562 peptide (40 mg/kg; 7 µmol/kg), Tat-peptide (20 mg/kg; 7 µmol/kg) or vehicle (saline), intraperitoneally for four days, once per day. On the last day, the mice were injected 30 minutes prior to extracting the tumor. For immunostaining, resected tumors were snap-frozen in isopentane submerged in liquid nitrogen and sectioned onto positive slides. Unstained frozen sections were fixed for 15 minutes in ice-cold acetone, dried, rehydrated in PBS and blocked in TBS containing 1% BSA, 10% goat serum and goat anti-mouse FAb (Jackson Immunoresearch, West Grove, PA) for 1 hour, followed by overnight (4°C) incubation with primary antibodies for phospho-Akt or phospho-Erk. After washing, Alexafluor 568-Goat anti-rabbit secondary antibodies (Invitrogen, USA) were incubated with the tissue for 1 hour at RT, followed by DAPI (Hoescht 33342, Molecular Probes, USA) staining. Staining was visualized using an Olympus MVX10 Macroview microscope with a 2X Apochromat lens with 5× zoom. Images were constructed into a montage using fluorescent tiling in the Olympus MicroSuite Biological Suite software.

For Western blot analysis, a 2 to 3 mm cross-sectional slice of the tumor was lysed in RIPA buffer by sonication and the resulting lysates were analyzed by Western blot following standard methods. Since samples contained both mouse and human tissue and cells, connective tissue and blood samples were taken from the mouse for comparison. The mouse samples have a high amount of total Erk (p44) and a negligible amount of basal phospho-Erk. In order to compare the level of phospho-Erk to the human tissue, the phospho-signal was normalized to a human tissue marker (human mitochondria).

### TCGA Data Analysis

We utilized protein expression level data provided through the TCGA for breast invasive carcinoma (BRCA) for total EGFR (antibody reference: EGFR-R-C) and phospho-EGFR (antibody reference: EGFR_pY1173-R-C) for 354 individuals. The values were normalized across the population such that the average is zero and the standard deviation is one for both the total and phosphor-EGFR expression. Two sets were obtained by separating individuals that had a normalized total EGFR level more than one standard deviation above the average but a normalized phosphor-EGFR level below one standard deviation above the average (20). Two individuals that had total EGFR levels more than 6.62 and 5.67 standard deviations away from the average level were excluded to give a remaining set of 320.

### Statistics

Plots and statistics were generated using Prism 5.0 (GraphPad Software, Inc.). Unless otherwise indicated, one-tailed, nonparametric Mann-Whitney tests (95% confidence interval) were used to determine if the mean values for each treatment condition were significantly different from control groups. P values are reported for each analysis in the figure legends, P values of 0.05 (or less) were considered significant.

## Supporting Information

Figure S1 (Related to Figure 1) **FAM-TE-66482 and FAM-Tat do not enter MDA-MB-231 cells. FAM-TE-64562 enters in SK-N-MC cells without any effect of EGF pretreatment.** Confocal images of overnight serum starved MDA-MB-231 cells before treatment (0 min) and treated for 90 minutes (**A**) with 5.0 µM FAM-TE-66482, (**B**) with 1.25 µM FAM-Tat or (C) with 2.5 µM FAM-Tat for 60 minutes. (**D**) SK-N-MC were serum starved overnight and treated with FAM-TE-64562 (1.5 µM) for 16 minutes. (**E**) NR6 cells MC were serum starved overnight and treated with FAM-TE-64562 (5 µM) for 20 minutes. All scale bars are 20 µm.
(TIF)

Figure S2 (Related to Figure 2) **Effect of TE-64562 on cell viability of different human cancer cell lines in the presence of 2.5% serum and RT-PCR for ERBB levels.** (**A**) The indicated cell line was serum starved overnight and treated with TE-64562 for 24 hours with varying concentrations (0.78 to 200 µM) of the peptide. Cell viability is measured as the percentage of viable cells after peptide treatment compared to untreated cells. Dose-response curves were generated and fitted in Prism 5.0 (GraphPad Software, Inc., USA). Error bars represent standard error from the mean of one experiment run in triplicate. Data are representative of at least two independent experiments. (**B**) RT-PCR of a selection of human cancer cell lines confirming the literature reports of ERBB expression levels. GAPDH was used as a house-keeping gene and all data were normalized to ERBB1, ERBB2, ERBB3 or ERBB4 expression in the MDA-MB-231 cell line. Data represent duplicate measurements from one experiment with error bars showing the standard deviation from the mean.
(TIF)

Figure S3 (Related to Figure 3) **Microscopy images and flow cytometry plots of MDA-MB-231 cells treated with TE-64562.** (**A**) MDA-MB-231 breast cancer cells were serum starved overnight then treated with 0 (control), 10 or 20 µM TE-64562 for 0.25, 0.5, 1, 3 or 24 hours and imaged. (**B**) MDA-MB-231 breast cancer cells were serum starved overnight then treated with 0 (control), 6 or 12 µM TE-64562 for 18 hours. Cells were stained with Annexin-V (Anx-V) and propidium iodide (PI). Staining is as follows: unstained viable cells (lower left quadrant; PI staining of non-viable cells (upper left quadrant); Anx-V plus PI staining of fully apoptotic and necrotic cells (upper right quadrant); Anx-V staining of apoptotic cells (lower right quadrant). The numbers in each quadrant represent the percentage of events/cells gated in each quadrant. Plots are representative of four experiments.
(TIF)

Figure S4 (Related to Figure 4) **Effects of TE-64562 on MDA-MB-231 xenograft tumors.** MDA-MB-231 xenograft tumors were grown in the subcutaneous flank region of nude mice. Treatments were commenced when tumors reached a size>100 mm^3^. (**A**) Mice were treated bi-weekly with the TE-64562 peptide (40 mg/kg; 7 µmol/kg), Tat peptide (20 mg/kg; 7 µmol/kg) or vehicle (saline), intraperitoneally. (**B**) Mice were treated as in (*A*) but with subcutaneous administration, proximal to the tumor site. Tumor size, measured bi-weekly, is plotted over time as an average for each treatment group. (**C**) Several H&E stained tumor slices from mice treated intraperitoneally for 17 days with TE-64562 or Saline and for 21 days with Tat, at the concentrations indicated above in (*A*). H&E images of tumors from mice treated for 14 to 52 days were (Tat, N = 6; Saline N = 8; TE-64562 N = 14) were quantified for the amount of viable tumor and necrotic/dead tissue and the averages ± S.D. are shown.
(TIF)

Figure S5 (Related to Figure 6) **The effect of TE-64562 on EGFR phosphorylation.** (**A–B**) Serum starved MDA-MB-231 cells were treated with the indicated concentration of TE-64562 or TKI for 30 minutes, followed by 10 ng/mL EGF for 10 minutes. (**A**) Phospho-EGFR (Y992) or (**B**) phospho-EGFR (Y1086) was analyzed by Western blot. (**C**) Cells were treated with 10 µM of TE-64562 or TKI for the 60 or 30 minutes, followed by 25 ng/mL EGF for 10 minutes. Phospho-EGFR (Y1045 and Y1173) was analyzed by Western blot. (**D**) Cells were treated with 20 µM of TE-64562 or 5 µM TKI for the indicated amounts of time, followed by 10 ng/mL EGF for 10 minutes. Phospho-EGFR (Y1068) was analyzed by Western blot. Data are representative of at least two experiments.
(TIF)

Figure S6 (Related to Figure 7) **Inhibition of p-Akt (S473) pAkt (T308) and p-Erk by TE-64562 in MDA-MB-231 cells and the inhibition of Akt and activation of JNK and p38 in MIA-PaCa-2 cells.** Serum starved MDA-MB-231 cells (A–E) or MIA-PaCa-2 cells (F) were treated with the indicated concentration of TE-64562, Tat (20.0 µM) or TKI (2.0 µM) for 30 minutes, followed by EGF for 10 minutes. (A–E) The presence of phospho-Akt (T308 or S473) and phospho-Erk (T202/T204) were analyzed by Western blot. (F) Serum starved MIA-PaCa-2 cells were analyzed for the presence of phospho-Akt (S473), phospho-Erk (T202/T204), phospho-JNK and phospho-p38 were analyzed by Western blot. MIA-PaCa-2 blots are representative of one of two independent experiments. Blots were stripped and re-probed with α-tubulin.
(TIF)

## References

[1] Pines G, Kostler WJ, Yarden Y (2010) Oncogenic mutant forms of EGFR: lessons in signal transduction and targets for cancer therapy. FEBS Lett 584: 2699–2706. 20388509 10.1016/j.febslet.2010.04.019 PMC2892754

[2] Takeuchi K, Ito F (2010) EGF receptor in relation to tumor development: molecular basis of responsiveness of cancer cells to EGFR-targeting tyrosine kinase inhibitors. FEBS J 277: 316–326. 19922467 10.1111/j.1742-4658.2009.07450.x

[3] Wheeler DL, Dunn EF, Harari PM (2010) Understanding resistance to EGFR inhibitors-impact on future treatment strategies. Nat Rev Clin Oncol 7: 493–507. 20551942 10.1038/nrclinonc.2010.97 PMC2929287

[4] Johnston JB, Navaratnam S, Pitz MW, Maniate JM, Wiechec E, et al (2006) Targeting the EGFR pathway for cancer therapy. Curr Med Chem 13: 3483–3492. 17168718 10.2174/092986706779026174

[5] De Roock W, Jonker DJ, Di Nicolantonio F, Sartore-Bianchi A, Tu D, et al (2010) Association of KRAS p.G13D mutation with outcome in patients with chemotherapy-refractory metastatic colorectal cancer treated with cetuximab. JAMA 304: 1812–1820. 20978259 10.1001/jama.2010.1535

[6] Vakiani E, Solit DB (2010) KRAS and BRAF: drug targets and predictive biomarkers. J Pathol 223: 219–229. 21125676 10.1002/path.2796

[7] Linardou H, Dahabreh IJ, Kanaloupiti D, Siannis F, Bafaloukos D, et al (2008) Assessment of somatic k-RAS mutations as a mechanism associated with resistance to EGFR-targeted agents: a systematic review and meta-analysis of studies in advanced non-small-cell lung cancer and metastatic colorectal cancer. Lancet Oncol 9: 962–972. 18804418 10.1016/S1470-2045(08)70206-7

[8] Pao W, Miller VA, Politi KA, Riely GJ, Somwar R, et al (2005) Acquired resistance of lung adenocarcinomas to gefitinib or erlotinib is associated with a second mutation in the EGFR kinase domain. PLoS Med 2: e73. 15737014 10.1371/journal.pmed.0020073 PMC549606

[9] Kobayashi S, Boggon TJ, Dayaram T, Janne PA, Kocher O, et al (2005) EGFR mutation and resistance of non-small-cell lung cancer to gefitinib. N Engl J Med 352: 786–792. 15728811 10.1056/NEJMoa044238

[10] Aifa S, Aydin J, Nordvall G, Lundstrom I, Svensson SP, et al (2005) A basic peptide within the juxtamembrane region is required for EGF receptor dimerization. Exp Cell Res 302: 108–114. 15541730 10.1016/j.yexcr.2004.08.032

[11] Macdonald-Obermann JL, Pike LJ (2009) The intracellular juxtamembrane domain of the epidermal growth factor (EGF) receptor is responsible for the allosteric regulation of EGF binding. J Biol Chem 284: 13570–13576. 19336395 10.1074/jbc.M109.001487 PMC2679458

[12] Thiel KW, Carpenter G (2007) Epidermal growth factor receptor juxtamembrane region regulates allosteric tyrosine kinase activation. Proc Natl Acad Sci U S A 104: 19238–19243. 18042729 10.1073/pnas.0703854104 PMC2148274

[13] Jura N, Endres NF, Engel K, Deindl S, Das R, et al (2009) Mechanism for activation of the EGF receptor catalytic domain by the juxtamembrane segment. Cell 137: 1293–1307. 19563760 10.1016/j.cell.2009.04.025 PMC2814540

[14] Martin-Nieto J, Villalobo A (1998) The human epidermal growth factor receptor contains a juxtamembrane calmodulin-binding site. Biochemistry 37: 227–236. 9425043 10.1021/bi971765v

[15] McLaughlin S, Smith SO, Hayman MJ, Murray D (2005) An electrostatic engine model for autoinhibition and activation of the epidermal growth factor receptor (EGFR/ErbB) family. J Gen Physiol 126: 41–53. 15955874 10.1085/jgp.200509274 PMC2266615

[16] Michailidis IE, Rusinova R, Georgakopoulos A, Chen Y, Iyengar R, et al (2011) Phosphatidylinositol-4,5-bisphosphate regulates epidermal growth factor receptor activation. Pflugers Arch 461: 387–397. 21107857 10.1007/s00424-010-0904-3 PMC3281421

[17] Sato T, Pallavi P, Golebiewska U, McLaughlin S, Smith SO (2006) Structure of the membrane reconstituted transmembrane-juxtamembrane peptide EGFR(622–660) and its interaction with Ca2+/calmodulin. Biochemistry 45: 12704–12714. 17042488 10.1021/bi061264m

[18] Red Brewer M, Choi SH, Alvarado D, Moravcevic K, Pozzi A, et al (2009) The juxtamembrane region of the EGF receptor functions as an activation domain. Mol Cell 34: 641–651. 19560417 10.1016/j.molcel.2009.04.034 PMC2719887

[19] Burgess AW (2008) EGFR family: structure physiology signalling and therapeutic targets. Growth Factors 26: 263–274. 18800267 10.1080/08977190802312844

[20] Bennasroune A, Fickova M, Gardin A, Dirrig-Grosch S, Aunis D, et al (2004) Transmembrane peptides as inhibitors of ErbB receptor signaling. Mol Biol Cell 15: 3464–3474. 15146055 10.1091/mbc.E03-10-0753 PMC452597

[21] Lofts FJ, Hurst HC, Sternberg MJ, Gullick WJ (1993) Specific short transmembrane sequences can inhibit transformation by the mutant neu growth factor receptor in vitro and in vivo. Oncogene 8: 2813–2820. 8104327

[22] Zwang Y, Yarden Y (2006) p38 MAP kinase mediates stress-induced internalization of EGFR: implications for cancer chemotherapy. EMBO J 25: 4195–4206. 16932740 10.1038/sj.emboj.7601297 PMC1570432

[23] Adachi S, Natsume H, Yamauchi J, Matsushima-Nishiwaki R, Joe AK, et al (2009) p38 MAP kinase controls EGF receptor downregulation via phosphorylation at Ser1046/1047. Cancer Lett 277: 108–113. 19138820 10.1016/j.canlet.2008.11.034

[24] Watanabe T, Otsu K, Takeda T, Yamaguchi O, Hikoso S, et al (2005) Apoptosis signal-regulating kinase 1 is involved not only in apoptosis but also in non-apoptotic cardiomyocyte death. Biochem Biophys Res Commun 333: 562–567. 15953587 10.1016/j.bbrc.2005.05.151

[25] deFazio A, Chiew YE, Sini RL, Janes PW, Sutherland RL (2000) Expression of c-erbB receptors, heregulin and oestrogen receptor in human breast cell lines. Int J Cancer 87: 487–498. 10918187

[26] Console S, Marty C, Garcia-Echeverria C, Schwendener R, Ballmer-Hofer K (2003) Antennapedia and HIV transactivator of transcription (TAT) “protein transduction domains” promote endocytosis of high molecular weight cargo upon binding to cell surface glycosaminoglycans. J Biol Chem 278: 35109–35114. 12837762 10.1074/jbc.M301726200

[27] Rusnak DW, Alligood KJ, Mullin RJ, Spehar GM, Arenas-Elliott C, et al (2007) Assessment of epidermal growth factor receptor (EGFR, ErbB1) and HER2 (ErbB2) protein expression levels and response to lapatinib (Tykerb, GW572016) in an expanded panel of human normal and tumour cell lines. Cell Prolif 40: 580–594. 17635524 10.1111/j.1365-2184.2007.00455.x PMC6495710

[28] Schoeberl B, Faber AC, Li D, Liang MC, Crosby K, et al (2010) An ErbB3 antibody, MM-121, is active in cancers with ligand-dependent activation. Cancer Res 70: 2485–2494. 20215504 10.1158/0008-5472.CAN-09-3145 PMC2840205

[29] Janmaat ML, Rodriguez JA, Jimeno J, Kruyt FA, Giaccone G (2005) Kahalalide F induces necrosis-like cell death that involves depletion of ErbB3 and inhibition of Akt signaling. Mol Pharmacol 68: 502–510. 15908515 10.1124/mol.105.011361

[30] Schaefer KL, Brachwitz K, Braun Y, Diallo R, Wai DH, et al (2006) Constitutive activation of neuregulin/ERBB3 signaling pathway in clear cell sarcoma of soft tissue. Neoplasia 8: 613–622. 16867224 10.1593/neo.06238 PMC1601931

[31] Verneris MR, Arshi A, Edinger M, Kornacker M, Natkunam Y, et al (2005) Low levels of Her2/neu expressed by Ewing’s family tumor cell lines can redirect cytokine-induced killer cells. Clin Cancer Res 11: 4561–4570. 15958642 10.1158/1078-0432.CCR-05-0157

[32] Pruss RM, Herschman HR (1977) Variants of 3T3 cells lacking mitogenic response to epidermal growth factor. Proc Natl Acad Sci U S A 74: 3918–3921. 302945 10.1073/pnas.74.9.3918 PMC431785

[33] Wang LM, Kuo A, Alimandi M, Veri MC, Lee CC, et al (1998) ErbB2 expression increases the spectrum and potency of ligand-mediated signal transduction through ErbB4. Proc Natl Acad Sci U S A 95: 6809–6814. 9618494 10.1073/pnas.95.12.6809 PMC22644

[34] Giovannini MG, Blitzer RD, Wong T, Asoma K, Tsokas P, et al (2001) Mitogen-activated protein kinase regulates early phosphorylation and delayed expression of Ca2+/calmodulin-dependent protein kinase II in long-term potentiation. J Neurosci 21: 7053–7062. 11549715 10.1523/JNEUROSCI.21-18-07053.2001 PMC6762991

[35] Ronai Z (1999) Deciphering the mammalian stress response - a stressful task. Oncogene 18: 6084–6086. 10557098 10.1038/sj.onc.1203175

[36] Rosette C, Karin M (1996) Ultraviolet light and osmotic stress: activation of the JNK cascade through multiple growth factor and cytokine receptors. Science 274: 1194–1197. 8895468 10.1126/science.274.5290.1194

[37] Chen P, Xie H, Sekar MC, Gupta K, Wells A (1994) Epidermal growth factor receptor-mediated cell motility: phospholipase C activity is required, but mitogen-activated protein kinase activity is not sufficient for induced cell movement. J Cell Biol 127: 847–857. 7962064 10.1083/jcb.127.3.847 PMC2120228

[38] Human Mammary Epithelial Cell (HMEC) Bank Website. Available: http://hmec.lbl.gov/mindex.html. Accessed 2012 Oct 17.

[39] Garbe JC, Holst CR, Bassett E, Tlsty T, Stampfer MR (2007) Inactivation of p53 function in cultured human mammary epithelial cells turns the telomere-length dependent senescence barrier from agonescence into crisis. Cell Cycle 6: 1927–1936. 17671422 10.4161/cc.6.15.4519

[40] Hammond SL, Ham RG, Stampfer MR (1984) Serum-free growth of human mammary epithelial cells: rapid clonal growth in defined medium and extended serial passage with pituitary extract. Proc Natl Acad Sci U S A 81: 5435–5439. 6591199 10.1073/pnas.81.17.5435 PMC391719

[41] Stampfer MR (1982) Cholera toxin stimulation of human mammary epithelial cells in culture. In Vitro 18: 531–537. 6288550 10.1007/BF02810076

[42] Stampfer MR, Yaswen P (2003) Human epithelial cell immortalization as a step in carcinogenesis. Cancer Lett 194: 199–208. 12757978 10.1016/s0304-3835(02)00707-3

