# Supplementary figures and images for: A Potential Peptide Therapeutic Derived from the Juxtamembrane Domain of the Epidermal Growth Factor Receptor
Source: PLoS One. 2012 Nov 15;7(11):e49702. doi: 10.1371/journal.pone.0049702 (PMC3499488; doi:10.1371/journal.pone.0049702)

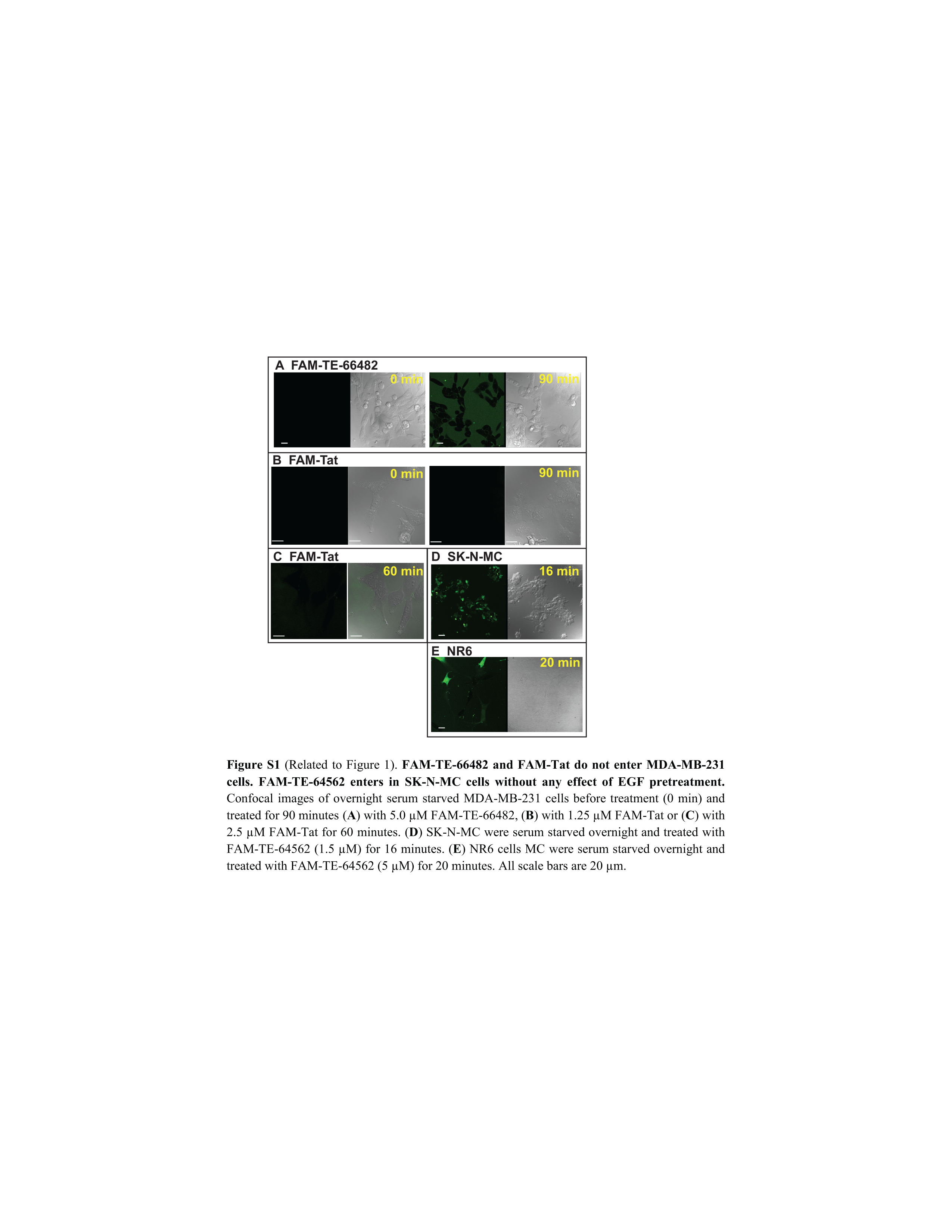

Supplement: Figure S1 (Related to Figure 1) — FAM-TE-66482 and FAM-Tat do not enter MDA-MB-231 cells. FAM-TE-64562 enters in SK-N-MC cells without any effect of EGF pretreatment. Confocal images of overnight serum starved MDA-MB-231 cells before treatment (0 min) and treated for 90 minutes (A) with 5.0 µM FAM-TE-66482, (B) with 1.25 µM FAM-Tat or (C) with 2.5 µM FAM-Tat for 60 minutes. (D) SK-N-MC were serum starved overnight and treated with FAM-TE-64562 (1.5 µM) for 16 minutes. (E) NR6 cells MC were serum starved overnight and treated with FAM-TE-64562 (5 µM) for 20 minutes. All scale bars are 20 µm. (TIF) [file pone.0049702.s001.tif]

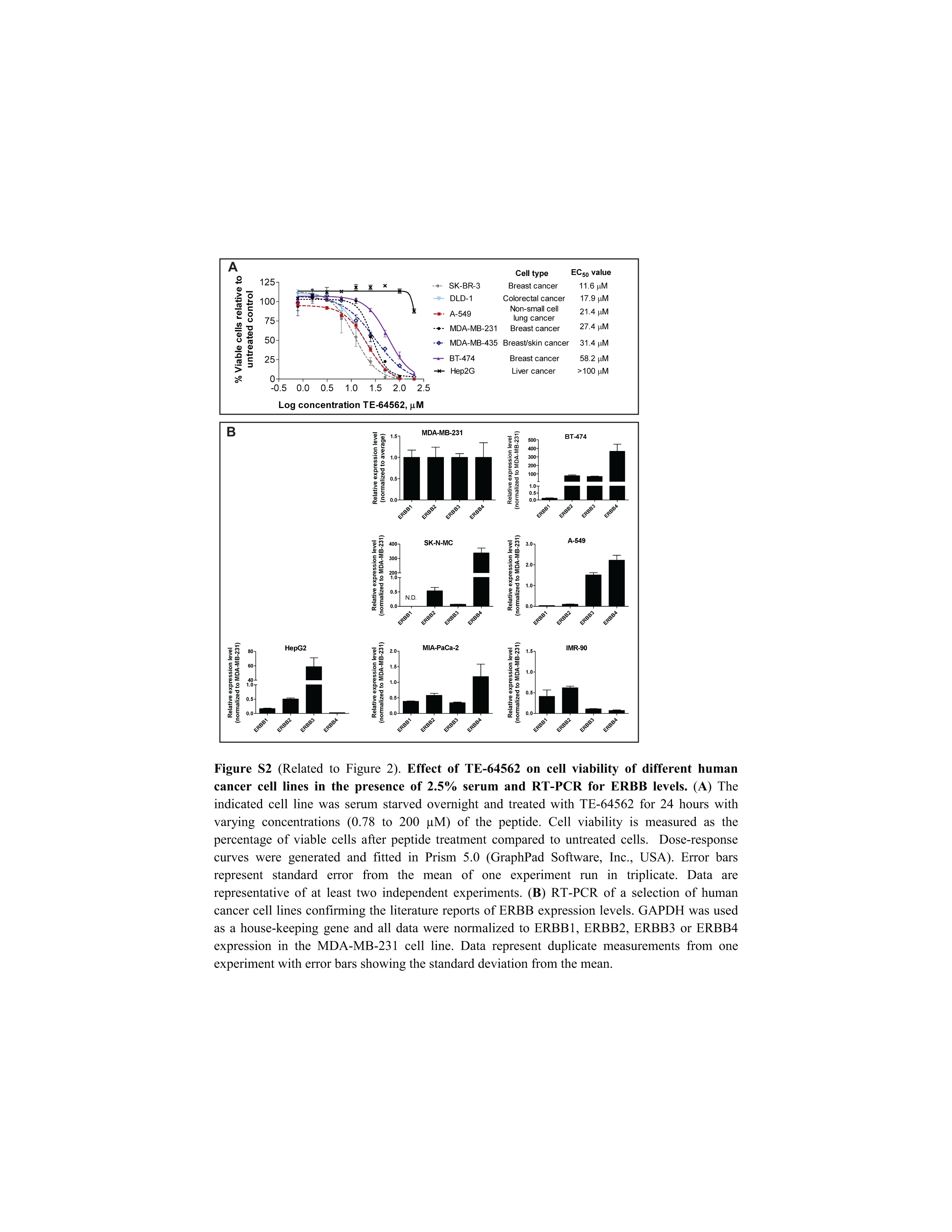

Supplement: Figure S2 (Related to Figure 2) — Effect of TE-64562 on cell viability of different human cancer cell lines in the presence of 2.5% serum and RT-PCR for ERBB levels. (A) The indicated cell line was serum starved overnight and treated with TE-64562 for 24 hours with varying concentrations (0.78 to 200 µM) of the peptide. Cell viability is measured as the percentage of viable cells after peptide treatment compared to untreated cells. Dose-response curves were generated and fitted in Prism 5.0 (GraphPad Software, Inc., USA). Error bars represent standard error from the mean of one experiment run in triplicate. Data are representative of at least two independent experiments. (B) RT-PCR of a selection of human cancer cell lines confirming the literature reports of ERBB expression levels. GAPDH was used as a house-keeping gene and all data were normalized to ERBB1, ERBB2, ERBB3 or ERBB4 expression in the MDA-MB-231 cell line. Data represent duplicate measurements from one experiment with error bars showing the standard deviation from the mean. (TIF) [file pone.0049702.s002.tif]

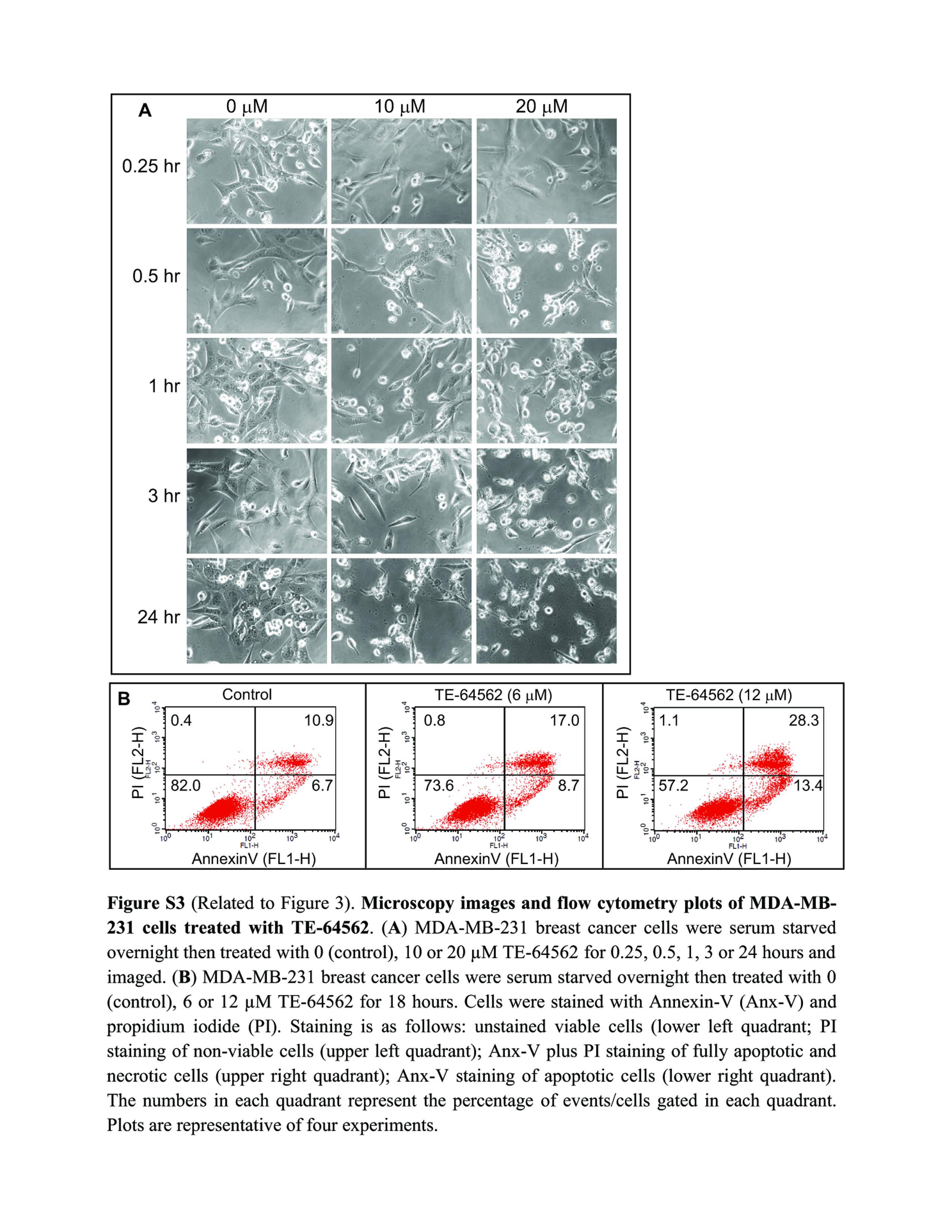

Supplement: Figure S3 (Related to Figure 3) — Microscopy images and flow cytometry plots of MDA-MB-231 cells treated with TE-64562. (A) MDA-MB-231 breast cancer cells were serum starved overnight then treated with 0 (control), 10 or 20 µM TE-64562 for 0.25, 0.5, 1, 3 or 24 hours and imaged. (B) MDA-MB-231 breast cancer cells were serum starved overnight then treated with 0 (control), 6 or 12 µM TE-64562 for 18 hours. Cells were stained with Annexin-V (Anx-V) and propidium iodide (PI). Staining is as follows: unstained viable cells (lower left quadrant; PI staining of non-viable cells (upper left quadrant); Anx-V plus PI staining of fully apoptotic and necrotic cells (upper right quadrant); Anx-V staining of apoptotic cells (lower right quadrant). The numbers in each quadrant represent the percentage of events/cells gated in each quadrant. Plots are representative of four experiments. (TIF) [file pone.0049702.s003.tif]

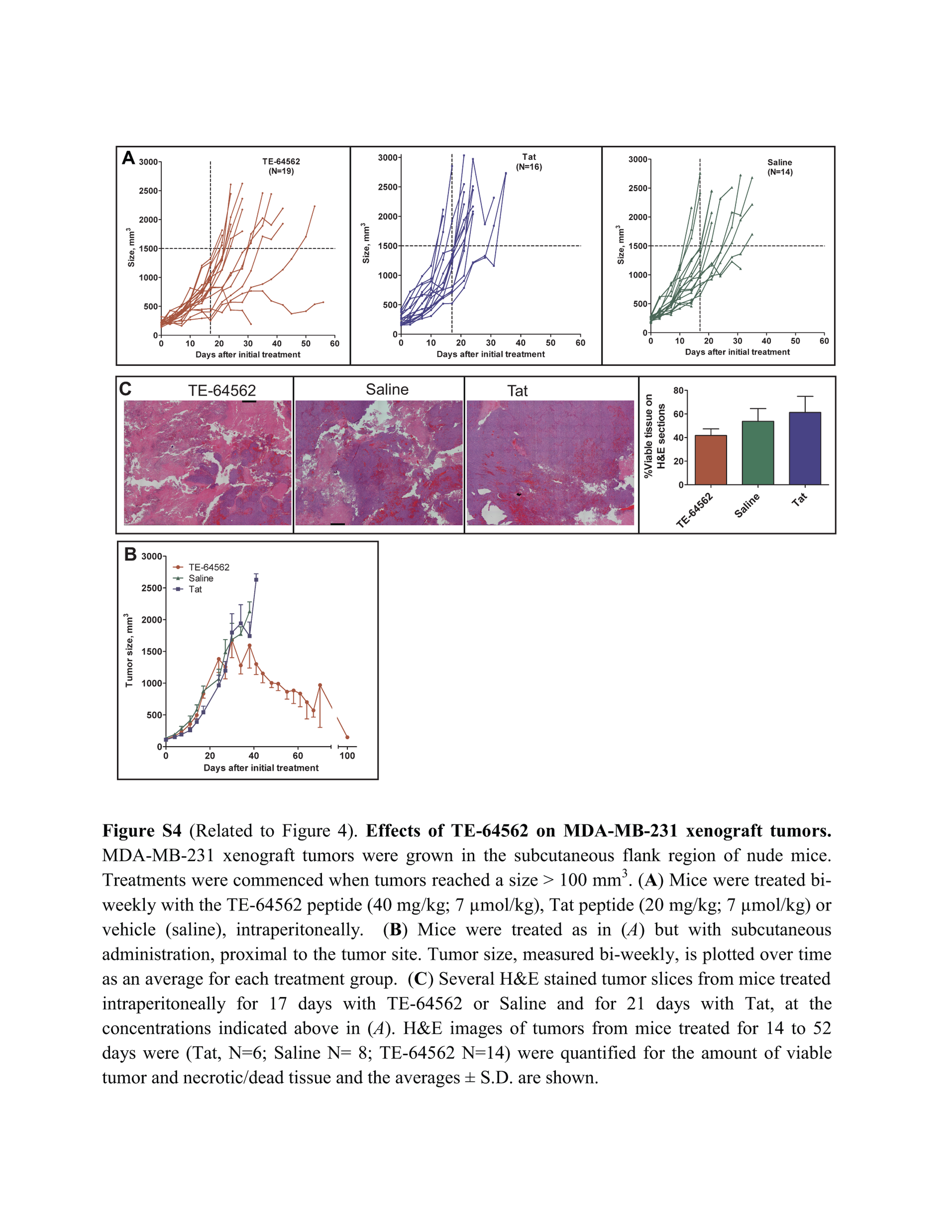

Supplement: Figure S4 (Related to Figure 4) — Effects of TE-64562 on MDA-MB-231 xenograft tumors. MDA-MB-231 xenograft tumors were grown in the subcutaneous flank region of nude mice. Treatments were commenced when tumors reached a size>100 mm3. (A) Mice were treated bi-weekly with the TE-64562 peptide (40 mg/kg; 7 µmol/kg), Tat peptide (20 mg/kg; 7 µmol/kg) or vehicle (saline), intraperitoneally. (B) Mice were treated as in (A) but with subcutaneous administration, proximal to the tumor site. Tumor size, measured bi-weekly, is plotted over time as an average for each treatment group. (C) Several H&E stained tumor slices from mice treated intraperitoneally for 17 days with TE-64562 or Saline and for 21 days with Tat, at the concentrations indicated above in (A). H&E images of tumors from mice treated for 14 to 52 days were (Tat, N = 6; Saline N = 8; TE-64562 N = 14) were quantified for the amount of viable tumor and necrotic/dead tissue and the averages ± S.D. are shown. (TIF) [file pone.0049702.s004.tif]

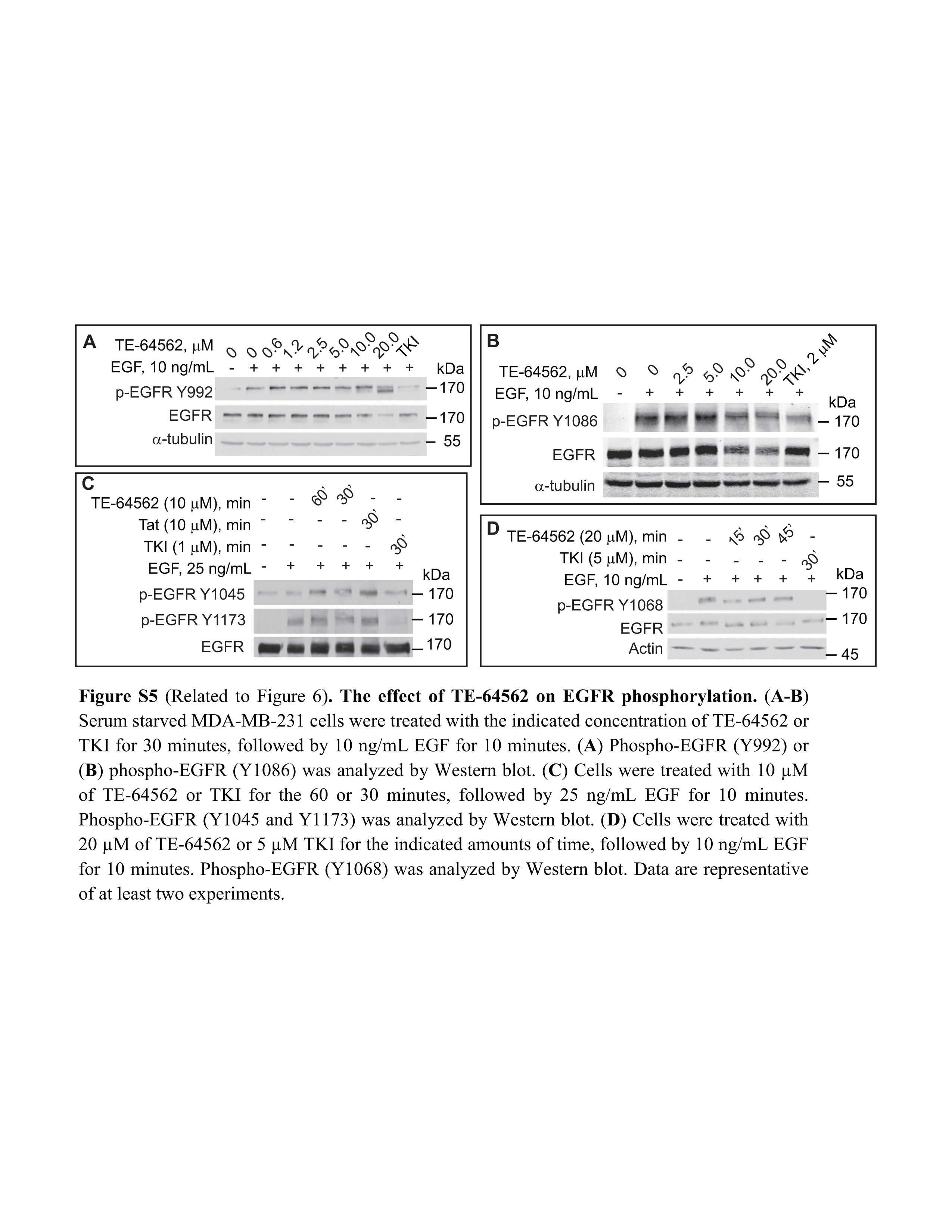

Supplement: Figure S5 (Related to Figure 6) — The effect of TE-64562 on EGFR phosphorylation. (A–B) Serum starved MDA-MB-231 cells were treated with the indicated concentration of TE-64562 or TKI for 30 minutes, followed by 10 ng/mL EGF for 10 minutes. (A) Phospho-EGFR (Y992) or (B) phospho-EGFR (Y1086) was analyzed by Western blot. (C) Cells were treated with 10 µM of TE-64562 or TKI for the 60 or 30 minutes, followed by 25 ng/mL EGF for 10 minutes. Phospho-EGFR (Y1045 and Y1173) was analyzed by Western blot. (D) Cells were treated with 20 µM of TE-64562 or 5 µM TKI for the indicated amounts of time, followed by 10 ng/mL EGF for 10 minutes. Phospho-EGFR (Y1068) was analyzed by Western blot. Data are representative of at least two experiments. (TIF) [file pone.0049702.s005.tif]

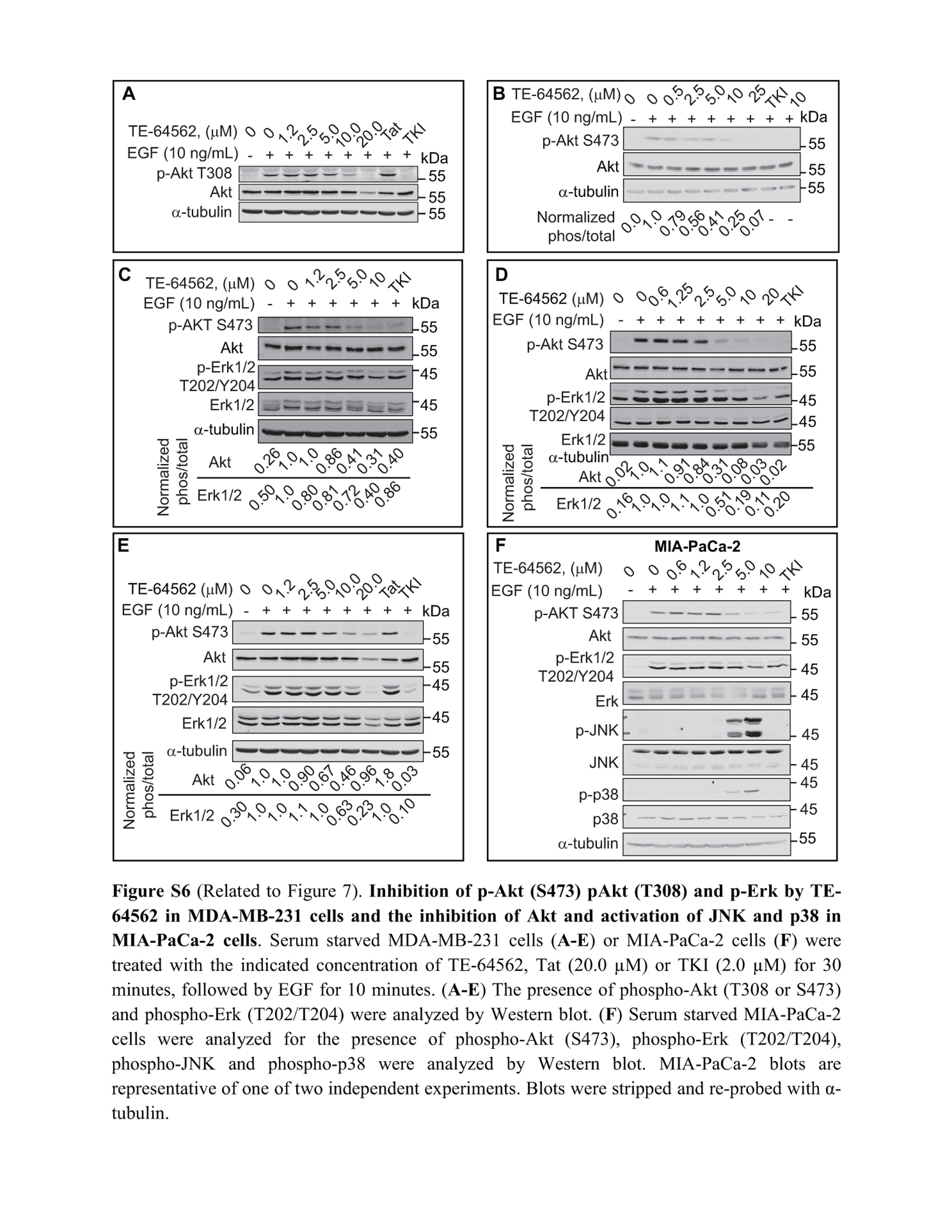

Supplement: Figure S6 (Related to Figure 7) — Inhibition of p-Akt (S473) pAkt (T308) and p-Erk by TE-64562 in MDA-MB-231 cells and the inhibition of Akt and activation of JNK and p38 in MIA-PaCa-2 cells. Serum starved MDA-MB-231 cells (A–E) or MIA-PaCa-2 cells (F) were treated with the indicated concentration of TE-64562, Tat (20.0 µM) or TKI (2.0 µM) for 30 minutes, followed by EGF for 10 minutes. (A–E) The presence of phospho-Akt (T308 or S473) and phospho-Erk (T202/T204) were analyzed by Western blot. (F) Serum starved MIA-PaCa-2 cells were analyzed for the presence of phospho-Akt (S473), phospho-Erk (T202/T204), phospho-JNK and phospho-p38 were analyzed by Western blot. MIA-PaCa-2 blots are representative of one of two independent experiments. Blots were stripped and re-probed with α-tubulin. (TIF) [file pone.0049702.s006.tif]
